# MLKL deficiency alleviates neuroinflammation and motor deficits in the α-synuclein transgenic mouse model of Parkinson’s disease

**DOI:** 10.1186/s13024-023-00686-5

**Published:** 2023-12-01

**Authors:** Lu Geng, Wenqing Gao, Hexige Saiyin, Yuanyuan Li, Yu Zeng, Zhifei Zhang, Xue Li, Zuolong Liu, Qiang Gao, Ping An, Ning Jiang, Xiaofei Yu, Xiangjun Chen, Suhua Li, Lei Chen, Boxun Lu, Aiqun Li, Guoyuan Chen, Yidong Shen, Haibing Zhang, Mei Tian, Zhuohua Zhang, Jixi Li

**Affiliations:** 1 State Key Laboratory of Genetic Engineering, Department of Neurology, Huashan Hospital and School of Life Sciences, MOE Engineering Research Center of Gene Technology, Shanghai Engineering Research Center of Industrial Microorganisms, Fudan University, Shanghai, 200438 China; 2 State Key Laboratory of Genetic Engineering, School of Life Sciences, Fudan University, Shanghai, 200438 China; 3 Insitute of Immunology, School of Medicine, Shanghai Jiaotong University, Shanghai, 200025 China; 4 Department of Neurology, Huashan Hospital and Institute of Neurology, Fudan University, Shanghai, 200040 China; 5 Division of Natural Science, Duke Kunshan University, Jiangsu, 215316 China; 6 Levi Regenerative Medicine Technologies, Zhuhai, 519085 China; 7 State Key Laboratory of Cell Biology, Shanghai Institute of Biochemistry and Cell Biology, Center for Excellence in Molecular Cell Science, Chinese Academy of Sciences, Shanghai, 200031 China; 8 CAS Key Laboratory of Nutrition, Metabolism and Food Safety, Shanghai Institute of Nutrition and Health, Chinese Academy of Sciences, Shanghai, 200031 China; 9 Human Phenome Institute, Fudan University, Shanghai, 200438 China; 10 Institute of Molecular Precision Medicine, Xiangya Hospital, Central South University, Changsha, 410078 Hunan China; 11 Department of Neurosciences, Hengyang Medical College, University of South China, Hengyang, 421001 Hunan China

**Keywords:** Parkinson’s disease, MLKL, Tg-*Mlkl*^*−/−*^ mice, Neuroinflammation, scRNA-seq

## Abstract

Parkinson’s disease (PD), one of the most devastating neurodegenerative brain disorders, is characterized by the progressive loss of dopaminergic neurons in the substantia nigra (SN) and deposits of α-synuclein aggregates. Currently, pharmacological interventions for PD remain inadequate. The cell necroptosis executor protein MLKL (Mixed-lineage kinase domain-like) is involved in various diseases, including inflammatory bowel disease and neurodegenerative diseases; however, its precise role in PD remains unclear. Here, we investigated the neuroprotective role of MLKL inhibition or ablation against primary neuronal cells and human iPSC-derived midbrain organoids induced by toxic α-Synuclein preformed fibrils (PFFs). Using a mouse model (Tg-*Mlkl*^*−/−*^) generated by crossbreeding the *SNCA* A53T synuclein transgenic mice with MLKL knockout (KO)mice, we assessed the impact of MLKL deficiency on the progression of Parkinsonian traits. Our findings demonstrate that Tg-*Mlkl*^*−/−*^ mice exhibited a significant improvement in motor symptoms and reduced phosphorylated α-synuclein expression compared to the classic A53T transgenic mice. Furthermore, MLKL deficiency alleviated tyrosine hydroxylase (TH)-positive neuron loss and attenuated neuroinflammation by inhibiting the activation of microglia and astrocytes. Single-cell RNA-seq (scRNA-seq) analysis of the SN of Tg-*Mlkl*^*−/*−^ mice revealed a unique cell type-specific transcriptome profile, including downregulated prostaglandin D synthase (PTGDS) expression, indicating reduced microglial cells and dampened neuron death. Thus, MLKL represents a critical therapeutic target for reducing neuroinflammation and preventing motor deficits in PD.

**Supplementary Information:**

The online version contains supplementary material available at 10.1186/s13024-023-00686-5.

## Introduction

Parkinson’s disease (PD) is the second most severe neurodegenerative disease, characterized by the degeneration of dopaminergic (DA) neurons in the substantia nigra pars compacta (SNpc) and a reduction of dopamine release in the striatum [1–3]. Despite extensive research, the pathogenesis of PD remains poorly understood. Genetic analyses have identified several genes involved in this process, including *SNCA*, *LRRK2*, and *PARKIN* [4]. The misfolded and aggregated forms of α-synuclein protein (denoted hereafter as α-Syn), which is encoded by the *SNCA* gene, have been implicated in the development of several neurodegenerative diseases, including PD, Parkinson disease dementia (PDD), dementia with Lewy bodies (DLB), incidental Lewy bodies disease (ILBD), and multiple system atrophy (MSA) [5–7]. Human SNCA consists of seven imperfect repeats with the consensus sequence KTKEGV, encompassing the lipid-binding domain [5]. Specific mutations in the *SNCA* gene have been linked to DLB, MS, and PD [8]. The A53T mutation has been found to accelerate the fibrillization of α-Syn and microtubule-associated protein tau in familial PD [9]. A transgenic mouse model expressing human A53T α-Syn has been shown to resemble human PD progression, exhibiting neuronal α-synucleinopathy, severe movement disorder, and features similar to their human counterparts in homozygotic mice aged 8 months or older [10].

While apoptosis is critical for the development of the central nervous system (CNS), necroptosis is the primary cell death involved in the adult CNS and neurodegenerative diseases [11]. Necroptosis, which is mediated by receptor-interacting protein kinase 1 (RIPK1 or RIP1), RIPK3 (or RIP3), and the pseudokinase MLKL, promotes necrotic cell death and neuroinflammation in Alzheimer’s disease (AD), amyotrophic lateral sclerosis (ALS), multiple sclerosis (MS), and PD [11–15]. Genetic deletion or pharmacological inhibition of necroptosis has been found to exert neuroprotective effects in multiple neurodegenerative diseases, including AD [16], ALS [17, 18], and MS [19]. However, the involvement of the necroptotic machinery (RIPK1/RIPK3/MLKL) in PD remains controversial. Studies have shown that treating animals with necrostatin-1 (Nec-1), an inhibitor of the RIPK1 kinase, does not affect 6-hydroxydopamine (6-OHDA)-dependent striatal denervation [20]. Moreover, α-Syn preformed fibrils (PFFs) have been found to induce neurotoxic astrocyte activation independent of MLKL and necroptosis [21]. It is also important to note that the 6-OHDA or MPTP-induced acute PD mouse models do not resemble the progressive Parkinson’s traits, which hinders their potential application in pharmacological intervention or clinical treatment [22, 23].

Here, we have generated a novel mouse model, designated as Tg-*Mlkl*^*−/−*^, through crossbreeding MLKL knockout mice and *SNCA* A53T mutation transgenic (denoted hereafter as A53T) mice. This new model closely mimics the progressive traits of PD. In vitro experiments revealed that inhibition of MLKL reduced cell death induced by 6-OHDA and TNF-α, or the toxic α-Syn preformed fibrils (PFFs). Moreover, depletion of MLKL resulted in an improvement in motor symptoms, a decrease in neuroinflammation, and a reduction in phosphorylated α-Syn expression in the substantia nigra (SN), cortex, and striatum regions of A53T transgenic mice. In addition, a single-cell RNA-seq analysis was performed to identify cell type-specific and disease-associated cellular subpopulations, providing a unique cellular perspective of transcriptional changes with MLKL deficiency in PD mice. Notably, upregulated synaptic-related neurons and downregulated microglia were identified in the SN region. Collectively, these findings suggest that inhibition of MLKL might represent a promising therapeutic strategy for PD.

## Results

### MLKL inhibition reduces cell death induced by PD stressors in vitro

**Fig. 1:**
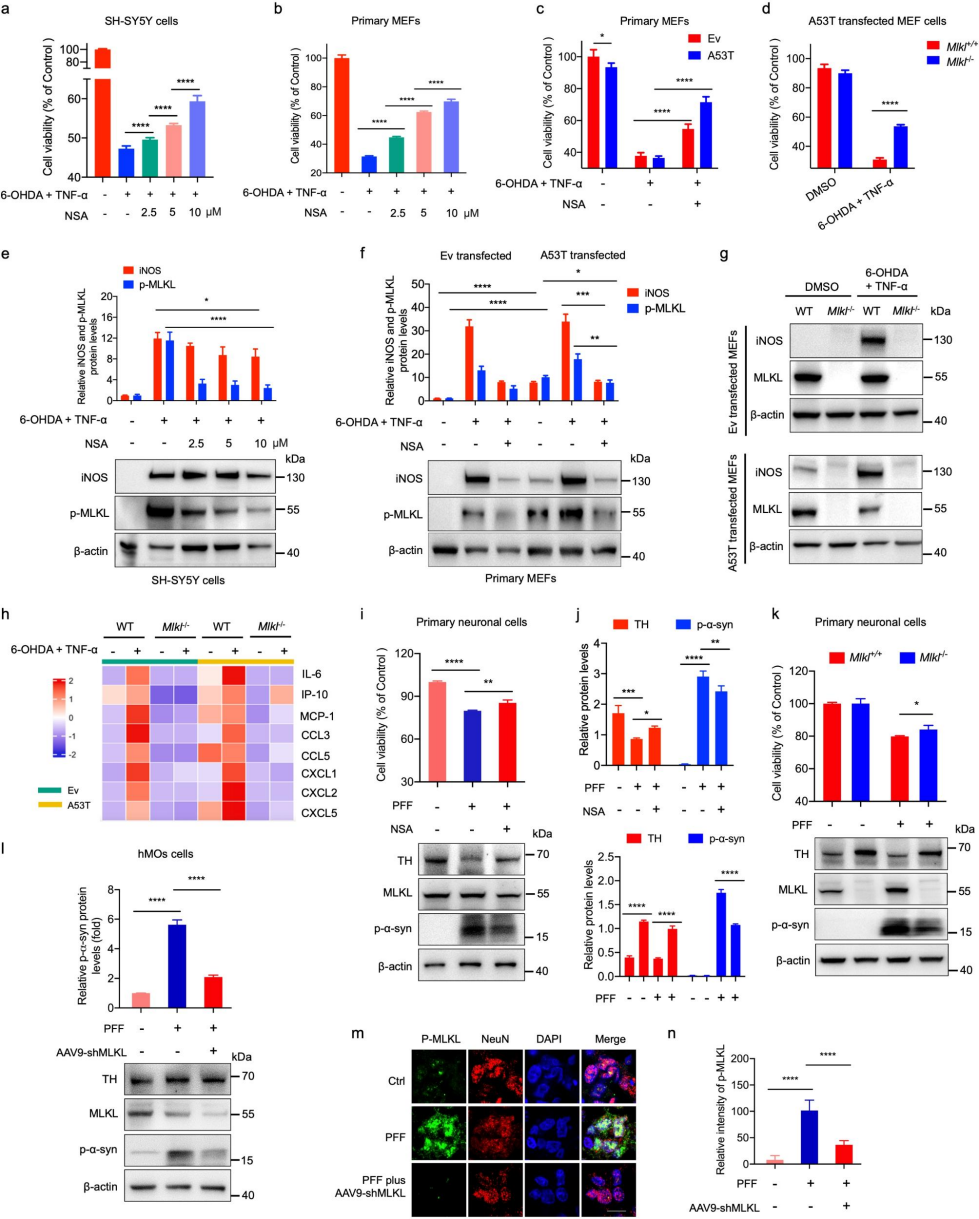
MLKL inhibition or deficiency decreased cell death in response to 6-OHDA plus TNF-α, or toxic α-Syn PFFs treatments. **a-b**. SH-SY5Y cells (a) or primary MEF cells (b) were treated with or without 6-OHDA plus TNF-α, followed by adding different concentrations of MLKL inhibitor NSA for 24 h. The cell viability was measured by the CCK8 assay and normalized to untreated cells. **c**. Primary MEF cells were transfected with GFP-tagged A53T synuclein (A53T) or empty vector (Ev). After 24 h, the transfected cells were treated with or without 6-OHDA, TNF-α, and NSA for another 24 h. **d**. *Mlkl*^*+/+*^ and *Mlkl*^*−/−*^ MEF cells were transfected with GFP-tagged A53T synuclein. After 24 h, the transfected cells were treated with or without 6-OHDA plus TNF-α for another 24 h. **e-f**. Cells were treated as described in **a** and **c**, respectively. Then western blot analysis was performed for inducible nitric oxide synthase (iNOS) and phosphorylated MLKL (p-MLKL). The quantification results were shown in the lower panels. **g-h**. *Mlkl*^*+/+*^ (WT) and *Mlkl*^*−/−*^ MEF cells were treated with GFP-tagged A53T synuclein (A53T) or empty vector (Ev). After 24 h, the transfected cells were treated with or without 6-OHDA plus TNF-α for another 24 h, followed by western blot analysis (**g**) and cytokine secretion measurement (**h**). **i-k**. Assessment of cell viability and protein expression in primary neuronal cells. Primary neuronal cells were treated with or without α-Syn preformed fibrils (PFFs) and NSA for 14 days followed by CCK8 assay and western blotting (**i**). *Mlkl*^*+/+*^ (WT) and *Mlkl*^*−/−*^ primary neuronal cells were treated with or without PFFs over a period of 14 days followed by the CCK8 assay and western blotting (**k**). Quantitative results from the experiments are presented in two panels. The upper panel displays the quantified data from the treatments in **i**, and the lower panel presents the quantification corresponding to the treatments in **k**. **l-n**. Human induced pluripotent stem cell (iPSC)-derived midbrain organoids (hMOs) were subjected to treatments with PFF, both with and without concurrent AAV9-shMLKL (to facilitate MLKL knockdown). Western blot analysis (**l**) and immunofluorescence staining (**m**) were employed to assess the treatments. The quantified expression levels of p-α-Syn are presented in the upper section of **l**, while the quantifications of p-MLKL intensity are illustrated in **n**. Scale bars, 10 μm. All data are representative of three independent experiments. The error bars represented the standard deviations (SD). * *p* < 0.05, ** *p* < 0.01, *** *p* < 0.001, **** *p* < 0.0001

To investigate the potential involvement of MLKL in Parkinson’s disease (PD) stress conditions, we performed experiments to evaluate the effect of MLKL inhibition on the cytotoxicity of 6-hydroxydopamine (6-OHDA), a neurotoxin commonly used to model PD, in human neuroblastoma cell line SH-SY5Y, and primary mouse embryonic fibroblasts (MEFs) (Fig. 1a and b and Fig. S1a-1d). SH-SY5Y cell line is frequently chosen in current PD research, and primary MEFs are sensitive to phospho-MLKL-triggered necroptotic cell death [24, 25]. Also, an MLKL inhibitor, necrosulfonamide (NSA), was employed with 6-OHDA plus TNF-α-induced cell death. We observed that TNF-α enhanced the sensitivity of both cell types to necrotic cell death in the presence of 6-OHDA (Fig. S1a-S1b). However, the addition of NSA significantly impaired this effect in a dose-dependent manner (Fig. 1a and b). Moreover, the phosphorylated MLKL (p-MLKL) and inducible nitric oxide synthase (iNOS), which are key markers of cell necroptosis, were highly expressed in 6-OHDA/TNF-α-treated cells; however, their expression was significantly reduced when treated with NSA (Fig. 1e and S1c-S1d).

As a high expression level of α-synuclein (α-Syn) has been reported to produce PD-like cellular and axonal pathologies in the nigrostriatal region [26, 27], we also investigated the effect of NSA on cell death induced by α-Syn aggregates under PD stress conditions. We transfected primary MEF cells with human A53T α-Syn-GFP and observed that NSA exhibited neuroprotective effects against 6-OHDA plus TNF-α, accompanied by downregulated expressions of p-MLKL and iNOS (Fig. 1c and f). Additionally, in A53T α-Syn-GFP-transfected MEF cells, knocking out *Mlkl* caused a significant decrease in 6-OHDA plus TNF-α-triggered cell death (Fig. 1d). Consistently, the expression of p-MLKL and iNOS were detectable in wild-type (WT) MEF cells, but not in *Mlkl*^*−/−*^ cells, even with 6-OHDA/TNF-α treatment (Fig. 1g). These results suggest that MLKL-mediated necroptosis may be positively correlated with oxidative stress responses.

Further enzyme-linked immunosorbent assay (ELISA) analyses revealed that 6-OHDA and TNF-α co-stimulation triggered the robust secretion of proinflammatory cytokines IL-6, IP-10, MCP-1, CCL3, CCL5, CXCL1, CXCL2, and CXCL5 (Fig. 1h). In line with these observations, the expression levels of chemokines, particularly IL-6 and MCP-1, were much higher in *Mlkl*^*+/+*^ MEF cells after 6-OHDA/TNF-α treatment but were lower in 6-OHDA/TNF-α-treated *Mlkl*^*−/−*^ MEF cells (Fig. 1h). These findings suggest that MLKL-mediated inflammatory signaling is highly associated with the 6-OHDA or α-Syn-induced PD model.

Further investigations into MLKL’s role in PD were conducted using primary neuronal cultures derived from the embryonic mesencephalon, a region that develops into the substantia nigra pars compacta (SNpc) during brain maturation. We utilized α-Syn preformed fibrils (PFFs) as a relevant neurotoxic stimulus for PD. Our findings revealed significant neuroprotective effects of NSA against PFFs, marked by decreased p-α-Syn and increased TH expression (Fig. 1i and j). Additionally, MLKL knockout markedly reduced PFFs-induced neuronal cell death (Fig. 1j and k). Correspondingly, p-α-Syn expression was significantly elevated in *Mlkl*^*+/+*^ primary neuronal cells upon α-Syn PFFs treatment, whereas reduced in PFFs-treated *Mlkl*^*−/−*^ primary neuronal cells (Fig. 1j and k).

To further elucidate the role of MLKL in PD pathology, we extended our investigations to human induced pluripotent stem cell (iPSC)-derived midbrain organoids (hMOs) subjected to the PFF-toxicity assays. The data revealed a pronounced upregulation of p-α-Syn expression in hMOs post-PFF treatments, which occurred concurrently with a reduction in MLKL expression and increased activation of p-MLKL within neuronal cells (Fig. 1l and n and S1e-S1f). Intriguingly, the targeted knockdown of MLKL significantly bolstered the resilience of hMOs to PFF-induced toxicity (Fig. 1l and n). Comparative analysis showed that hMOs treated with PFFs in conjunction with AAV9-shMLKL displayed a substantial decline in p-α-Syn levels and neuronal p-MLKL expression (Fig. 1l and n). These findings underscore that the lack or suppression of MLKL expression imparts a protective effect, mitigating the cellular impacts of diverse PD-related stressors in murine and human models.

### MLKL deficiency ameliorates motor symptoms in the A53T transgenic mice

**Fig. 2:**
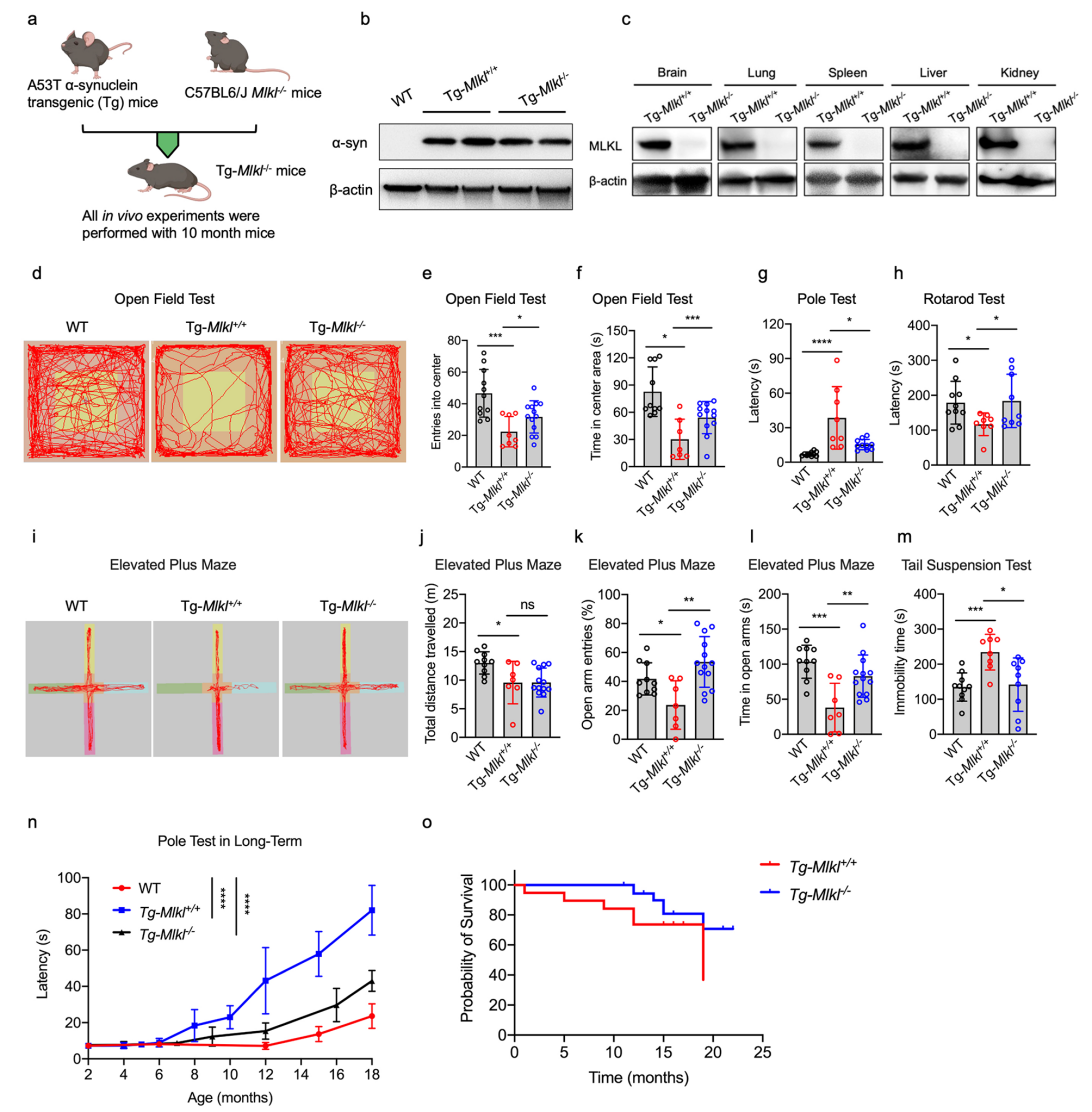
MLKL deficiency improves motor capability in homozygous A53T synuclein transgenic (Tg) mice. Wild-type (WT, n = 10), Tg*-Mlkl*^*+/+*^ (n = 7), and Tg*-Mlkl*^*−/−*^ mice (n = 13) around 10–12 months old were examined for different motor activities as described in the Methods section. **a**. Schematic of the crossbreeding between A53T transgenic mice (expressing mutant human α-synuclein) and MLKL KO mice. **b-c**. Immunoblotting analysis of the protein expression levels of α-synuclein (α-Syn) and MLKL in multiple tissues from the Tg*-Mlkl*^*+/+*^ and Tg*-Mlkl*^*−/−*^ mice. **d-f**. The autonomous trajectory map of WT, Tg*-Mlkl*^*+/+*^, and Tg*-Mlkl*^*−/−*^ mice in the open field test. Tg*-Mlkl*^*−/−*^ mice exhibited more entries (**e**) and times (**f**) in the center region than Tg*-Mlkl*^*−/−*^ mice. **g**. The average time required for the WT, Tg*-Mlkl*^*+/+*^, and Tg*-Mlkl*^*−/−*^ mice to descend the pole. **h**. WT, Tg*-Mlkl*^*+/+*^, and Tg*-Mlkl*^*−/−*^ mice were scored for latency to fall on the accelerating rotarod. **i-l**. Performance of mice in the elevated plus-maze test (EPMT). **i**. The autonomous trajectory maps of WT, Tg*-Mlkl*^*+/+*^, and Tg*-Mlkl*^*−/−*^ mice in the EPMT were recorded. **j**. The total distance traveled in 5 min was shown. The Tg*-Mlkl*^*−/−*^ mice had more entries into the open arms (**k**) and spent more time in the anxiety-provoking open arms (**l**) than the Tg*-Mlkl*^*−/−*^mice. **m**. Detection of depression with the tail suspension test. The Tg*-Mlkl*^*−/−*^ mice showed a significant reduction in the duration of immobility compared with the control mice. **n**. Long-term pole test results from 2–18 months, comparing WT, Tg-*Mlkl*^*+/+*^, and Tg-*Mlkl*^*−/−*^ mice. **o**. Survival curves for Tg-*Mlkl*^*+/+*^ (n = 19) and Tg-*Mlkl*^*−/−*^ (n = 37) mice. All data are representative of three independent experiments. The error bars represented the standard deviations (SD). * *p* < 0.05, ** *p* < 0.01, *** *p* < 0.001, **** *p* < 0.0001, ns, no significance

The inhibition or deficiency of MLKL was observed to reduce cell death and proinflammatory cytokine secretion in the presence of 6-OHDA-induced PD traits, as depicted in Fig. 1. Next, we investigated the role of MLKL in motor capability and anxiety-like behaviors in progressive PD using Tg*-Mlkl*^*−/−*^ mice generated by crossbreeding *Mlkl*^*−/−*^ mice with human A53T α-Syn transgenic (denoted hereafter as Tg) mice (Fig. 2a) [10, 28]. Western blot analysis of different tissues of the Tg*-Mlkl*^*−/−*^ mice revealed the absence of MLKL and overexpression of human A53T α-Syn protein (Fig. 2b and c). As the Tg-*Mlkl*^*+/+*^ mice exhibit typical PD characteristics, including abnormal motor activities at around ten months old [10], we subjected the Tg*-Mlkl*^*−/−*^ mice and control groups (WT and Tg*-Mlkl*^*+/+*^ mice) to perform behavioral tests (Fig. 2d-m).

The Tg*-Mlkl*^*−/−*^ mice showed similar locomotor activities in the open field with the WT mice, while more active than the Tg*-Mlkl*^*+/+*^ mice (Fig. 2d). Moreover, the Tg*-Mlkl*^*−/−*^ mice exhibited reduced anxiety-like behavior compared to the Tg-*Mlkl*^*+/+*^ mice, as evidenced by their increased entries and longer time spent in the center zone, indicating a decrease in avoidance of the center (Fig. 2d and f). Additionally, the pole test revealed that the Tg-*Mlkl*^*−/−*^ mice exhibited a significant decrease in the time taken to reach the pole base compared to the control Tg-*Mlkl*^*+/+*^ group (Fig. 2g), demonstrating an improvement in movement disorder caused by striatal dopamine depletion.

Furthermore, the Tg-*Mlkl*^*−/−*^ mice exhibited significantly reduced behavioral deficits observed in the Tg-*Mlkl*^*+/+*^ mice as assessed by the accelerating rotarod test (Fig. 2h). The elevated plus-maze test (EPMT) showed that the Tg-*Mlkl*^*−/−*^ mice spent a higher percentage of arm entries and time in the open arm compared to the Tg-*Mlkl*^*+/+*^ mice, as depicted in Fig. 2i L; however, there was no significant difference observed in the total distance traveled between the two groups (Fig. 2i and j). Finally, the tail suspension test was utilized to evaluate the depressive-like symptoms of mice, where the Tg-*Mlkl*^*−/−*^ mice exhibited decreased immobility times when compared to the Tg-*Mlkl*^*+/+*^ mice (Fig. 2m). Long-term motor function assessment using the pole test highlighted that Tg-*Mlkl*^*−/−*^ mice, especially at 18 months, took significantly less time to reach the pole base compared to Tg-*Mlkl*^*+/+*^ mice (Fig. 2n). Survival analysis revealed a prolonged lifespan in Tg-*Mlkl*^*−/−*^ mice compared to Tg-*Mlkl*^*+/+*^ mice, with a median survival time of 19 months (Fig. 2o). In conclusion, our results suggest that *Mlkl* knockout can effectively improve motor capabilities and alleviate depressive symptoms in Tg-*Mlkl*^*+/+*^ mice exhibiting PD.

### MLKL deficiency protects dopaminergic neurons in A53T transgenic mice

**Fig. 3:**
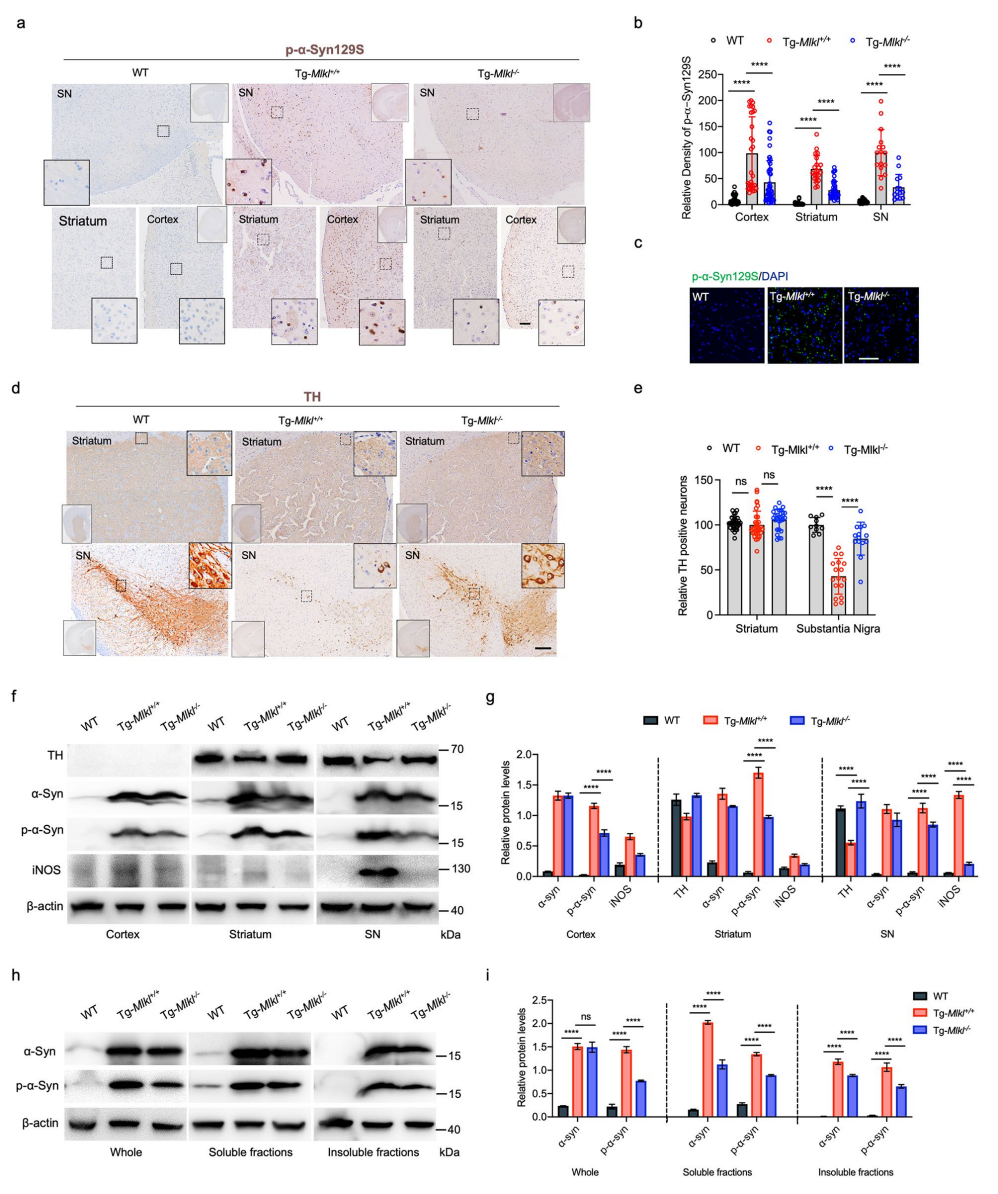
MLKL deficiency protects dopaminergic neuron loss and decreases hyperphosphorylated α-synuclein in the A53T transgenic mice. **a**. Representative immunostaining for phosphorylated α-Syn (p-α-Syn129S) in the cortex, striatum, and substantia nigra (SN) regions of WT, Tg*-Mlkl*^*+/+*^ and Tg*-Mlkl*^*−/−*^ mice. Scale bars, 100 μm. The whole-brain sections were shown in the top right corner. The solid rectangles were zoomed from the selected dashed rectangles in the cortex, striatum, and SN regions, respectively. The quantification results of p-α-Syn129S are shown in **b**. **c**. Representative immunofluorescence image of the striatum region in the frozen brain sections of WT, Tg*-Mlkl*^*+/+*^, and Tg*-Mlkl*^*−/−*^ mice staining with DAPI (blue, denoting the nuclear signal) and anti-p-α-Syn antibody (green). Scale bars, 50 μm. **d**. Dopaminergic neurons were determined by tyrosine hydroxylase (TH) staining. Representative TH immunostaining images of the cortex and SN sections from WT, Tg*-Mlkl*^*+/+*^, and Tg*-Mlkl*^*−/−*^ mice were shown in **d**. The whole-brain sections were shown in the bottom left corner. The solid rectangles were zoomed from the selected dashed rectangles in the striatum and SN regions. Scale bars, 100 μm. **e.** Quantification of the total number of TH-positive cells in the entire striatum and SN regions, corresponding with **d**. **f-g**. Representative Western blot results of the expression levels of TH, α-Syn, p-α-Syn, and iNOS in the cortex, striatum, and SN sections of the WT, Tg*-Mlkl*^*+/+*^ and Tg*-Mlkl*^*−/−*^ mice (**f**). Quantifications of the expressions for TH, α-Syn, p-α-Syn, and iNOS were shown in **g**. **h-i**, Western blot results displaying α-Syn and p-α-Syn levels in whole brain tissue (Whole), soluble fractions, and insoluble fractions from WT, Tg-*Mlkl*^*+/+*^, and Tg-*Mlk*^*l−/−*^ mice. The soluble fraction contains cytoplasmic proteins, while the insoluble fraction includes membrane-bound proteins, organelle-associated proteins, and nuclear proteins. Quantifications of the results for α-Syn and p-α-Syn were shown in **i**. All data are representative of three independent experiments. The error bars represented the standard deviations (SD). ** *p* < 0.01, *** *p* < 0.001, **** *p* < 0.0001, ns, no significance

To investigate whether MLKL deficiency improves motor capability by regulating α-Syn function, we conducted immunoblotting, immunofluorescence, and immunohistochemical staining for phosphorylated α-Syn at serine 129 (p-α-Syn129S), a specific pathological form associated with α-Syn aggregation in PD [29]. Our results demonstrated that p-α-Syn129S was present in high abundance in the cortex, striatum, and substantia nigra regions of Tg-*Mlkl*^*+/+*^ mice; however, they markedly decreased in the corresponding regions of Tg-*Mlkl*^*−/−*^ mice (Fig. 3a and b). Additionally, immunofluorescence staining confirmed that MLKL deficiency significantly reduced phosphorylated α-Syn inclusions in the striatum region of the A53T transgenic mice (Fig. 3c). In humans with Parkinson’s disease, the substantial loss of dopaminergic (DA) neurons in the SN is closely related to motor dysfunction [30]. Although there was no significant difference in dopaminergic neurodegeneration in the striatum among the three mouse groups (Fig. 3d and e), we observed a higher level of TH-positive neurons in the SN of Tg-*Mlkl*^*−/−*^ mice and WT mice, but not in Tg-*Mlkl*^*+/+*^ mice (Fig. 3d and e). Furthermore, the DA neurons exhibited different morphologies in the SN regions of Tg-*Mlkl*^*+/+*^ and Tg-*Mlkl*^*−/−*^ mice. DA neurons in the pars compacta of the SN in Tg-*Mlkl*^*−/−*^ mice exhibited a high density of TH-positive fibers and contained a denser cell mass than those in Tg-*Mlkl*^*+/+*^ mice (Fig. 3d). Immunoblot analysis also demonstrated that TH accumulation was significantly elevated, accompanied by a remarkable reduction of p-α-Syn and iNOS in the cortex, striatum, and/or SN of Tg-*Mlkl*^*−/−*^ mice (Fig. 3f g). Furthermore, we segregated the whole brain tissues of the three mouse cohorts into soluble and insoluble components for Immunoblot analysis. This revealed that the whole brain tissues of Tg-*Mlkl*^*+/+*^ mice exhibited significantly higher levels of α-Syn and p-α-Syn compared to WT mice (Fig. 3h and i). Notably, the deletion of the *Mlkl* gene substantially diminished the p-α-Syn expression in Tg-*Mlkl*^*+/+*^ mouse brains (Fig. 3h and i). Additionally, in Tg-*Mlkl*^*−/−*^ mice, the levels of p-α-Syn were consistently lower in soluble and insoluble fractions than in Tg-*Mlkl*^*+/+*^ mice (Fig. 3h and i).

Given that the homozygous line M83^+/+^ A53T mice naturally manifest a pronounced motor phenotype between 8 and 16 months linked with the accumulation of α-Syn inclusions in areas such as the spinal cord, brain stem, thalamus, periaqueductal gray, mesencephalon (surrounding the substantia nigra), and dorsal cochlear nucleus (DCN) [10], we further investigated the expression of p-α-Syn129S and NeuN (a neuron-specific nuclear protein) in the spinal cord and DCN of Tg-*Mlkl*^*+/+*^ and Tg-*Mlkl*^*−/−*^ mice. Our findings illustrate that *Mlkl* gene knockout significantly reduces the expression of p-α-Syn129S in the spinal cord, mesencephalon, and DCN brain regions of Tg mice, while notably augmenting NeuN expression in the spinal cord (Fig. S2a-2b), consistent with above results (Fig. 3a and e). Additionally, double immunofluorescence staining of p-α-Syn129S and NeuN in the cortical area of both mouse groups indicated that *Mlkl* gene knockout genuinely diminishes the accumulation of p-α-Syn129S in neuronal cells (Fig. S2c). Consequently, these findings suggest that MLKL-mediated signaling is intricately associated with dopaminergic neurodegeneration and α-Syn aggregation in mice.

### MLKL deficiency attenuates neuroinflammation in A53T transgenic mice

**Fig. 4:**
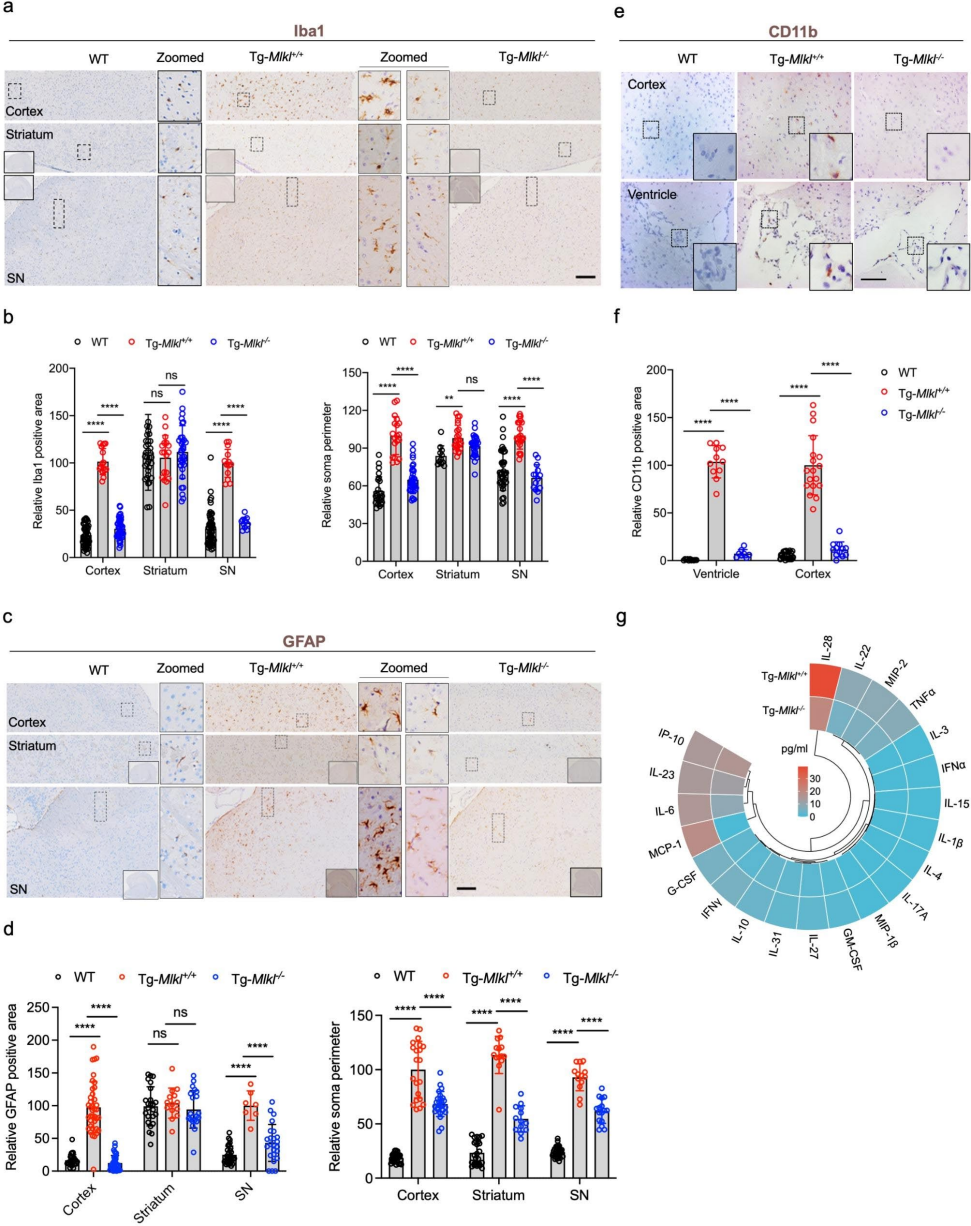
MLKL deficiency attenuates neuroinflammation in the A53T transgenic mice. **a-b**. Representative immunostaining for the microglial marker Iba1 in the cortex, striatum, and SN regions of WT, Tg*-Mlkl*^*+/+*^, and Tg*-Mlkl*^*−/−*^ mice (**a**). The quantification results of Iba1 intensity and relative soma perimeter are shown in **b**. The whole-brain sections were shown in solid rectangles. The dashed rectangle regions were zoomed in and shown in the middle. Scale bars, 200 μm. **c-d**. Immunohistochemistry staining and quantification of GFAP expression. Representative images of GFAP immunoreactivity from WT, Tg*-Mlkl*^*+/+*^, and Tg*-Mlkl*^*−/−*^ mice were shown in **c**, and the according quantification results were shown in **d**. **e-f.** Representative immunostaining for CD11b in the cortex and ventricle of WT, Tg*-Mlkl*^*+/+*^, and Tg*-Mlkl*^*−/−*^ mice (**e**). The quantification results of CD11b are shown in **f**. Scale bars, 50 μm. The whole-brain sections were shown in solid rectangles. The dashed rectangle regions were zoomed in and shown in the middle. Scale bars, 200 μm. **g**. The heat map depicted the average baseline serum cytokine concentration for the Tg*-Mlkl*^*+/+*^ (n = 3) and Tg*-Mlkl*^*−/−*^ mice (n = 3). All data are representative of three independent experiments. The error bars represented the standard deviations (SD). ** *p* < 0.01, *** *p* < 0.001, **** *p* < 0.0001, ns, no significance

Microglia activation, as evidenced by increased expression of Iba-1, can indirectly indicate a neuronal abnormality in A53T transgenic (Tg-*Mlkl*^*+/+*^) mice. To investigate this, we assessed the expression of Iba1 and found that *Mlkl* knockout significantly reduced Iba1 immunoreactivity in the cortex and substantia nigra (SN) of Tg-*Mlkl*^*+/+*^ mice (Fig. 4a and b). Additionally, MLKL deficiency resulted in a significant decrease in the expression of CD11b, a surface receptor that is upregulated on activated microglia and is involved in the neuroinflammatory response in the brain [31], in the cortex and ventricle regions of Tg-*Mlkl*^*+/+*^ mice (Fig. 4e and f).

The pathological marker, glial fibrillary acidic protein (GFAP), was prominently accumulated in the cortex and SN regions of Tg-*Mlkl*^*+/+*^ mice. In contrast, it was significantly decreased in the cortex and SN regions of Tg-*Mlkl*^*−/−*^ mice (Fig. 4c and d). Notably, microglia and astrocytes showed enlarged somas in Tg-*Mlkl*^*+/+*^ mice, indicating morphological activation; however, microglia and astrocytes shrank when *Mlkl* was knocked out (Fig. 4b and d). These findings indicate that MLKL deficiency significantly attenuated microglia and astrocyte activation, ameliorating Parkinson’s symptoms in A53T transgenic (Tg-*Mlkl*^*+/+*^) mice.

**Fig. 5:**
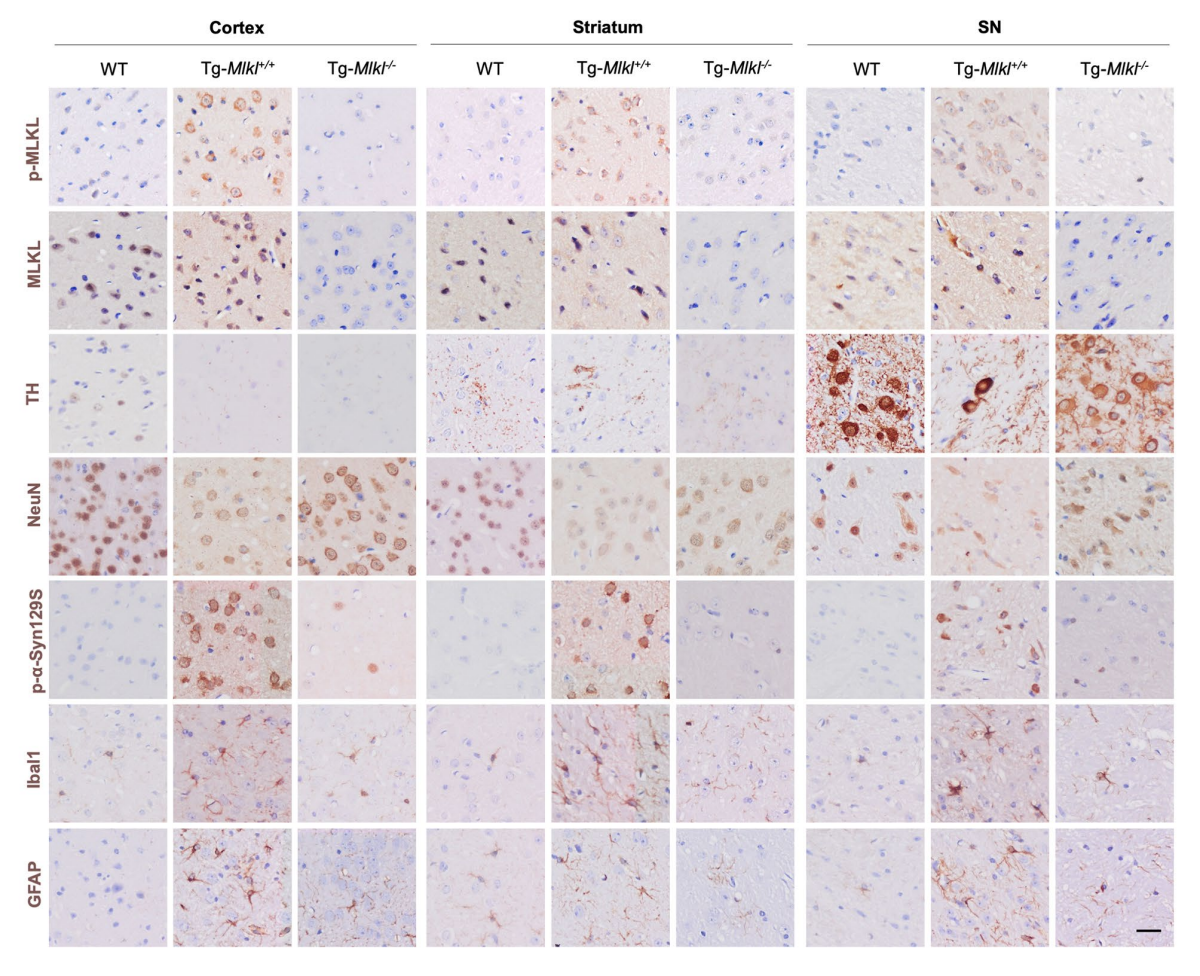
MLKL deficiency reduces MLKL and p-MLKL levels in A53T transgenic mice. Seven consecutive tissue sections were obtained from the same brain region (cortex, striatum, and SN) of individual WT, Tg-*Mlkl*^*+/+*^, and Tg-*Mlkl*^*−/−*^ mice. These sections underwent IHC to determine the expression levels of MLKL, p-MLKL, NeuN, TH, p-α-Syn, Iba1, and GFAP. Scale bars, 25 μm. All data are representative of three independent experiments

Moreover, as MLKL-mediated necroptosis exacerbates multiple neurodegenerative diseases by triggering cell death and neuroinflammation [32], we evaluated the production of multiple serum cytokines using the ELISA method. Our results demonstrated that many proinflammatory cytokines, including IL6 and MCP-1, were significantly reduced in Tg-*Mlkl*^*−/−*^ mice compared to Tg-*Mlkl*^*+/+*^ mice (Fig. 4g), which was consistent with the aforementioned results (Fig. 1h). In addition, we performed IHC analyses on six consecutive brain sections from each mouse group. The analyses showed that, in comparison to WT mice, Tg-*Mlkl*^*+/+*^ mice exhibited a notable increase in phosphorylated MLKL (p-MLKL) within the cortex, striatum, and substantia nigra (Fig. 5). This increase coincided with reduced levels of TH and NeuN, and elevated levels of p-α-Syn, Iba1, and GFAP. In contrast, *Mlkl* knockout in Tg mice significantly lowered p-MLKL expression in these brain regions, accompanied by a reduction in p-α-Syn, Iba1, and GFAP, and an upregulation of TH and NeuN expressions (Fig. 5). These results imply a significant association between MLKL expression in the mouse brain and the extent of neuronal damage and neuroinflammatory activity.

### Single-cell RNA sequencing (scRNA-seq) analysis reveals the upregulated synaptic-related neurons and downregulated microglia in the SN region of the Tg-*Mlkl*^*−/−*^ mice

**Fig. 6:**
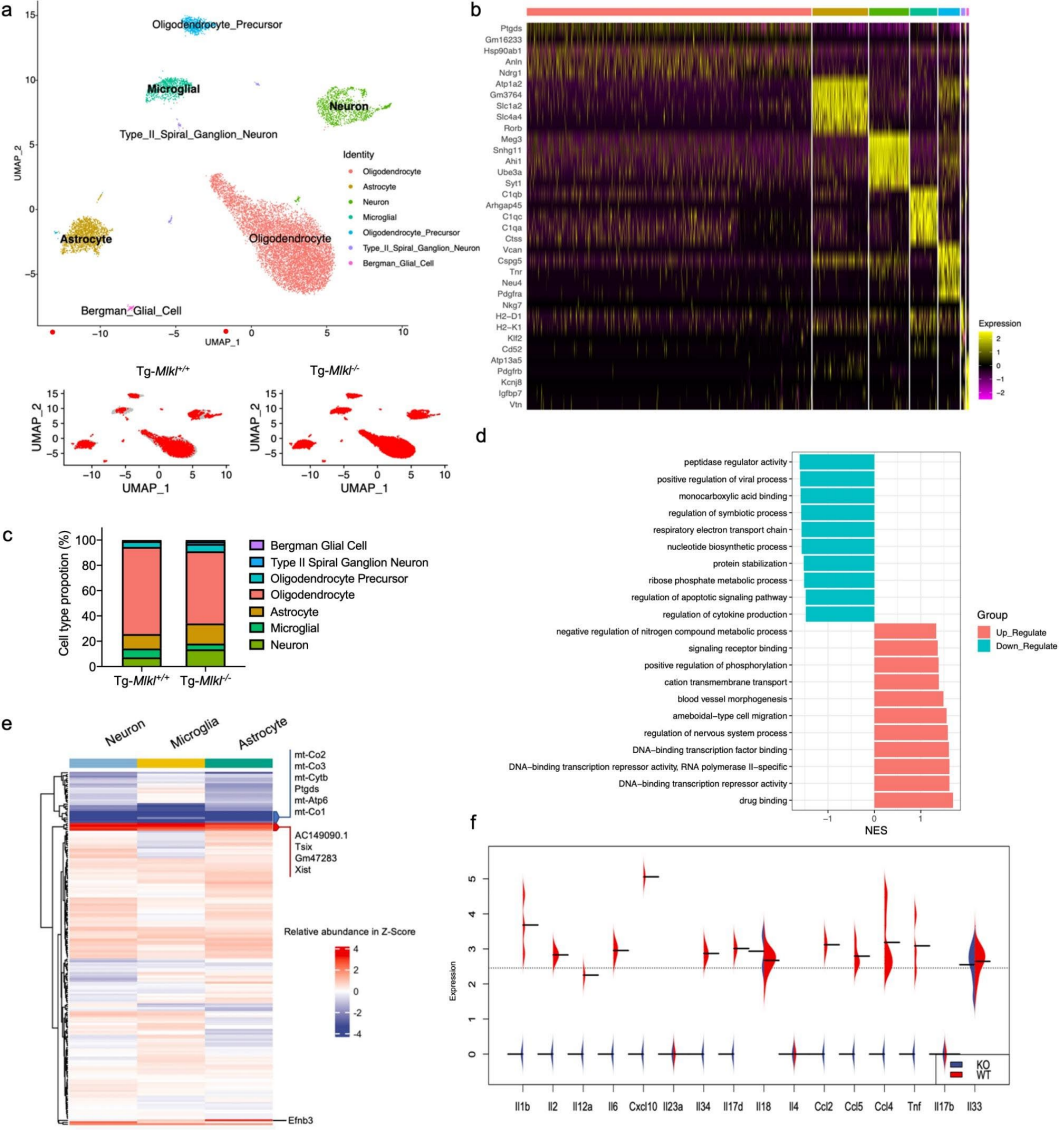
Single-cell RNA-seq analysis reveals the upregulated synaptic-related neurons and downregulated microglia in the substantia nigra region of the Tg*-Mlkl*^*−/−*^ mice. **a**. UMAP visualization showing the clustering of single nuclei colored by cell types (upper) or individuals (bottom) in the SN regions of the Tg*-Mlkl*^*+/+*^ and Tg*-Mlkl*^*−/−*^ mice. **b**. Heatmap showing the top 5 markers for each of the 7 clusters. **c**. The proportions of different types of cells in the Tg*-Mlkl*^*+/+*^ and Tg*-Mlkl*^*−/−*^ mice. **d**. Heatmap depicting the z-scores of the common differential expressed signature genes in neuron, microglia, and astrocyte clusters. Representative genes were highlighted on the right side. The colors represent the cluster’s mean expression (transcripts per million). **e**. Top biological pathways enriched for DEGs were identified across neuron cells in the SN region. **f**. Beanplot showed different cytokine genes expression in microglia of the Tg*-Mlkl*^*+/+*^ (WT) and Tg*-Mlkl*^*−/−*^ (KO) mice

To investigate the role of MLKL in advanced PD, we conducted scRNA-seq on nuclei isolated from substantia nigra regions of Tg-*Mlkl*^*−/−*^ and Tg-*Mlkl*^*+/+*^ mice (Fig. S3). In the SN region of Tg-*Mlkl*^*+/+*^ mice (n = 3), we generated 3,563 single nuclei gene expression profiles, with a median of 405 genes and 100,993 transcripts per nucleus. For the SN region of Tg-*Mlkl*^*−/−*^ mice (n = 3), we generated 8,466 single nuclei gene expression profiles, with a median of 497 genes and 43,481 transcripts per nucleus (Fig. S4a-S4b). In addition, we utilized Uniform Manifold Approximation and Projection (UMAP) visualization to separate nuclei into distinct clusters (Fig. 6a). Next, we annotated these clusters using cell-type-specific markers to identify oligodendrocytes (e.g., *Ptgds*, *Gm16233*, *Anln*, *Ndrg1*, and *Gng11*), oligodendrocyte precursor cells (e.g., *Vcan*, *Cspg5*, *Thr*, *Neu4*, and *Pdgfra*), astrocytes (e.g., *Atp1a2*, *Gm3764*, *Slc4a4*, *Slc1a2*, and *Rorb*), neurons (e.g., *Meg3*, *Snhg11*, *Ahi1*, *Ube3a*, and *Syt1*), Bergman glial cells (e.g., *Atp13a5*, *Pdgfrb*, *Kcnj8*, *Igfbp7*, and *Vtn*), type II spiral ganglion neurons (e.g., *H2-D1*, *H2-K1*, *Kif2*, *Cd52*, and *Ly6c1*), and microglia (e.g., *C1qb*, *Arhgap45*, *C1qc*, *C1qa*, and *Ctss*) (Fig. 6b).

We observed significant differences in the cluster sizes between the two groups, with a marked increase in the proportions of neurons and astrocytes in the Tg-*Mlkl*^*−/−*^ mice, while oligodendrocytes and microglia were more frequent in the Tg-*Mlkl*^*+/+*^ mice (Fig. 6a and c). Additionally, we identified three clusters (neuron, microglia, and astrocyte) that exhibited both cell-type-specific and common gene expression patterns between the Tg-*Mlkl*^*−/−*^ and Tg-*Mlkl*^*+/+*^ mice (Fig. 6e).

Differential expression of genes (DEGs) was identified in neuron, microglia, and astrocyte clusters between Tg-*Mlkl*^*−/−*^ and Tg-*Mlkl*^*+/+*^ mice, followed by gene ontology (GO) term enrichment analysis of biological processes (Fig. 6d and e and S5a-S5b). Notably, Tg-*Mlkl*^*−/−*^ neuronal cells exhibited upregulation of nervous system processes (e.g., *Efnb3*) and downregulation of nitrogen compound metabolic processes (e.g., *Tsix*) (Fig. 6d and e). Furthermore, downregulated genes in Tg-*Mlkl*^*−/−*^ neuronal cells were enriched in functions related to cytokine production and apoptotic signaling pathways, indicating reduced inflammation and cell death (Fig. 6d and f). Similarly, microglia from Tg-*Mlkl*^*−/−*^ mice showed upregulation of DEGs related to neurogenesis and downregulation of DEGs that are positive regulators of inflammation and secretion (Fig. S5a). Additionally, upregulated genes were enriched in neuron projection development and morphogenesis, while DEGs related to innate immune response were downregulated in Tg-*Mlkl*^*−/−*^ astrocytes (Fig. S5b).

Furthermore, by employing 15 markers (Fig. S6b), we differentiated the neuron cluster into a dopaminergic neuron cluster, designated as Thpos, and the remaining cells formed the Thneg cluster (Fig. S6a). Notably, in Tg-*Mlkl*^*−/−*^ mice, the neuronal cells showed a higher ratio of Thpos compared to Tg-*Mlkl*^*+/+*^ mice (Fig. S6c). Upon conducting GO analysis on these differential genes, it was observed that Thpos cells from Tg-*Mlkl*^*−/−*^ mice demonstrated an upregulation in DEGs associated with the regulation of signaling receptor activity and GABAergic synapse (Fig. S6d). Conversely, genes showing a decrease were predominantly involved in pathways related to Parkinson’s disease, oxidative phosphorylation, ribosome, and mitochondrial functions (Fig. S6d).

**Fig. 7:**
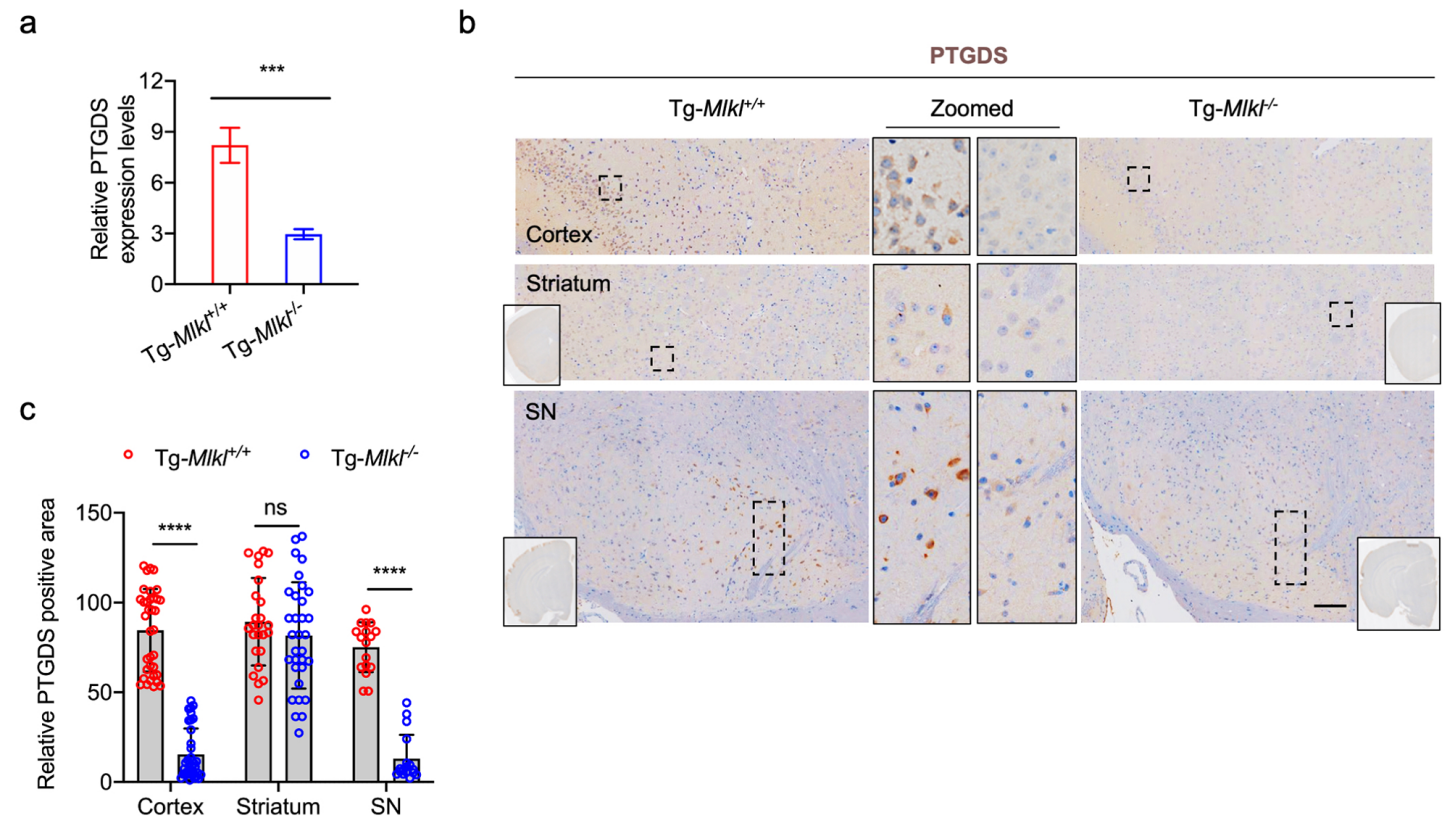
PTGDS mRNA and protein expression between Tg*-Mlkl*^*+/+*^ and Tg*-Mlkl*^*−/−*^ mice. **a**. The qRT-PCR results of PTGDS in the SN region of Tg-*Mlkl*^*+/+*^ and Tg-*Mlkl*^*−/−*^ mice. **b-c**. Representative immunostaining for PTGDS in the cortex, striatum, and SN regions of Tg-*Mlkl*^*+/+*^ and Tg-*Mlkl*^*−/−*^ mice (**b**). The whole-brain sections were shown in solid rectangles. The dashed rectangle regions were zoomed in and shown in the middle. Scale bars, 200 μm. The quantification results of PTGDS are shown in **c**. All data are representative of three independent experiments. The error bars represented the standard deviations (SD). *** *p* < 0.001, **** *p* < 0.0001, ns, no significance

The differentially expressed genes (DEGs) within the neuron, microglia, and astrocyte clusters were combined to identify shared up-regulated and down-regulated genes. A heatmap displaying the clustering analysis of these 180 common DEGs is presented in Fig. 6e. Among them, the *mt-Co1*, *mt-Co2*, *mt-Co3*, *mt-Cytb*, *mt-Atp6*, *Ptgds*, *AC149090.1*, *Tsix*, *Gm47283*, and *Xist* genes exhibited the highest expression changes (Fig. 6e). Notably, five out of the ten DEGs (*mt-Co1*, *mt-Co2*, *mt-Co3*, *mt-Cytb*, and *mt-Atp6*) are critical components of the mitochondrial electron transport chain (ETC) and mitochondrial respiratory chain [33, 34]. Mitochondrial dysfunction has long been implicated in the pathogenesis of PD [35], and several studies have identified mutations in *mt-Co2, mt-Co2*, *mt-Co3*, and *mt-Atp6* associated with various clinical brain disorders [36]. Glutathione-independent prostaglandin D synthase (PTGDS), a prostaglandin involved in pain and sleep, was also identified as a unique blood-based signature capable of differentiating between idiopathic PD patients and controls [37]. A recent study demonstrated that *PTGDS* was upregulated in PD patients and could serve as an optimal biomarker for PD diagnosis [37]. In our case, quantitative real-time polymerase chain reaction (qRT-PCR) was conducted, confirming a 2.8-fold decrease in PTGDS mRNA expression in Tg-*Mlkl*^*−/−*^ compared to Tg-*Mlkl*^*+/+*^ mice (Fig. 7a). IHC also confirmed that MLKL deficiency increased PTGDS protein levels in the cortex, striatum, and SN regions of the A53T transgenic (Tg) mice (Fig. 7b and c).

Given the observed reduction of numerous proinflammatory cytokines in Tg-*Mlkl*^*−/−*^ mice (Fig. 4g), we conducted a systematic examination of 16 PD-associated cytokines [38, 39] within the identified microglia cluster. Our findings revealed that 11 cytokine genes, including *Il1b*, *Il2*, *Il12a*, *Il6*, *Cxcl10*, *Il34*, *Il17d*, *Ccl2*, *Ccl5*, *Ccl4*, and *Tnf*, were downregulated in Tg-*Mlkl*^*−/−*^ mice (Fig. 6f). These results provide a detailed snapshot of global and cell-type-specific changes in gene expression and functional processes associated with MLKL deficiency in PD progression.

### The subcluster-specific analysis identifies specific and functional cell transcriptomics subclusters after MLKL deficiency in the A53T transgenic mice

**Fig. 8:**
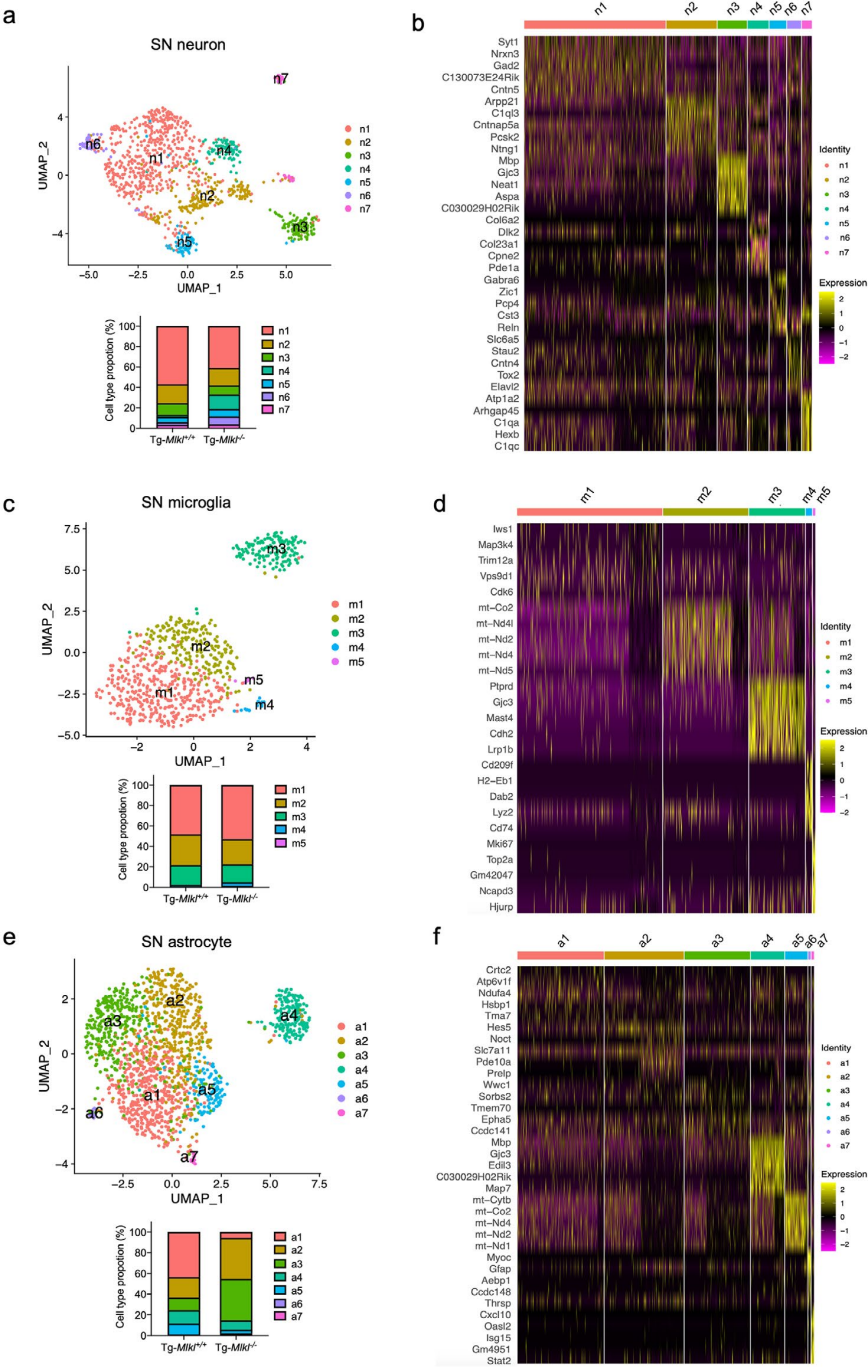
The subcluster-specific analysis identifies specific and functional cell subclusters after MLKL deficiency in the A53T transgenic mice. **a**, **c**, **e**. UMAP visualization of subclusters of neurons (**a**), microglia (**c**), and astrocytes (**e**). The frequency distribution of each subcluster between the Tg*-Mlkl*^*+/+*^ and Tg*-Mlkl*^*−/−*^ mice was shown at the bottom. **b**, **d**, **f**. Significantly enriched genes in the neurons (**b**), microglia (**d**), and astrocyte subclusters (**f**)

We utilized the Seurat algorithm to partition neurons, microglia, and astrocytes into subclusters based on their transcriptional characteristics. Through an unbiased analysis, we identified seven neuronal clusters, five microglial clusters, and seven astrocyte clusters, each enriched with specific functional categories and characterized by distinct molecular markers (Fig. 8a and f). Interestingly, our findings revealed that MLKL deficiency did not significantly affect the proportion of microglial subclusters. In contrast, it led to the segregation of neurons and astrocytes into different clusters in Tg-*Mlkl*^*−/−*^ mice compared to Tg-*Mlkl*^*+/+*^ mice (Fig. 8a and f).

In particular, the n4 and n6 subclusters of Tg-*Mlkl*^*−/−*^ mice showed higher proportions compared to those of Tg-*Mlkl*^*+/+*^ mice (Fig. 8a). These subclusters were enriched with genes associated with tissue growth and repair (*Col6a2* and *Col23a1*), membrane proteins regulating trafficking, adipogenesis, or potassium absorption (*Dlk2*, *Cpne2*, and *Atp12a*), and neuronal RNA transporting (*Stau2* and *Elavl2*) (Fig. 8b). Additionally, gene expression changes and enrichment analysis of the n4 subcluster in Tg-*Mlkl*^*−/−*^ mice revealed an association with the response to cellular stress, including the cellular response to DNA damage stimulus, starvation, and nutrient levels (Fig. S7a). Furthermore, the upregulated genes of the n7 cluster in Tg-*Mlkl*^*−/−*^ mice were linked to the negative regulation of the nitrogen compound metabolic process, consistent with our western blot data (Fig. 8b and S7a).

While only one microglial subcluster m4 exhibited a higher frequency in Tg-*Mlkl*^*−/−*^ mice, most subcluster-specific genes across three microglial subclusters (m1-m3) were downregulated and enriched for processes including structural molecule activity, peptide metabolic and biosynthetic processes, and ribosome subunit (Fig. 8c and d and S7b). Regarding astrocyte subclusters, both a2 and a3 clusters were highly abundant in Tg-*Mlkl*^*−/−*^ mice and expressed genes related to signal transduction and memory performance (*Pde10a* and *Wwc1*), biogenesis of mitochondrial ATP synthase (*Tmem70*), neurogenesis and synaptogenesis (*Hes5* and *Epha5*) (Fig. 8e and f). Notably, the cystine-glutamate exchanger, *Slc7a11*, which could inhibit ferroptosis and cell necrosis, and suppress inflammatory and oxidative responses, was upregulated in clusters a2 and a3 of Tg-*Mlkl*^*−/−*^ mice (Fig. 8e and f). Furthermore, the a3 subcluster of astrocytes in Tg-*Mlkl*^*−/−*^ mice showed an upregulation of genes associated with the negative regulation of cytokine production, suggesting a non-inflammatory phenotype for this subcluster (Fig. S7c).

## Discussion

Although the necroptotic machinery RIPK1-RIPK3-MLKL is involved in PD progression and cell necroptosis promotes cell death and neuroinflammation [20, 22], their crosstalking remains controversial. Previous studies on drug-inducible acute PD mouse models have shown that inhibition of RIPK1 kinase activity by Nec-1 can protect dopaminergic neurons and attenuate neurotoxicity [11]. Similarly, genetic ablation of RIPK3 or pharmacological inhibition of RIPK1 in the 6-OHDA-induced PD mouse model has been found to decrease dopaminergic neuron degeneration and improve motor performance [20, 40]. Also, necroptosis-induced neuroinflammation might occur at the onset of PD and lead to neuronal dopaminergic degeneration [32]. However, all the acute animal model studies lacked predictive values for identifying neurodegenerative or neuroprotective agents, and failed to resemble the progressive traits of PD, especially the presence of Lewy bodies and Lewy neurites [41].

Lewy bodies are the hallmark pathological feature of PD, composed primarily of fibrillar α-Syn, which is known to interfere with normal cellular processes and contribute to the death of dopaminergic neurons in SN. In addition, genetic mutations in the SNCA gene, which encodes the α-Syn protein, particularly the A53T mutation, have been linked to the formation of abnormal aggregates in the brain of PD patients and an increased risk of developing the disease.

To address these issues, we created a new mouse model (Tg-*Mlkl*^*−/−*^) using SNCA A53T transgenic mice to investigate the role of the cell necroptosis executor MLKL in PD. This model was designed to replicate several critical features of human PD, including the formation of Lewy bodies, the loss of dopaminergic neurons in SN, microglial inflammation, and motor dysfunction. Additionally, we generated a comprehensive data atlas for single-cell transcriptomic analysis of the SN region in the PD mouse model, which provides a unique resource to understand cellular heterogeneity and define functional changes at a single-cell resolution under MLKL deficiency. Importantly, by doing this, we showed that targeting MLKL could alleviate neuronal inflammatory death, reduce neuroinflammation, and improve motor behaviors in the PD mouse model.

Our in vivo data demonstrated that the knockout of MLKL significantly reduced dopaminergic neuron loss and alleviated motor dysfunction in Tg-*Mlkl*^*+/+*^ mice. Additionally, the formation of p-α-Syn129S inclusions and microglia activation were dramatically reduced after *Mlkl* deficiency (Figs. 3 and 4). Furthermore, proinflammatory cytokines, including IL6 and MCP1, were reduced in 6-OHDA plus TNFα treated neuron cells and in the A53T transgenic mice when MLKL was knocked out (Figs. 1 and 4). Although previous studies have suggested that inflammasome signaling may contribute to the formation of α-Syn aggregates [42–44], our data revealed that α-Syn released from degenerating neurons stimulates microglia, leading to microglia activation and cytokine secretion (Figs. 3, 4 and 5). These data suggest that α-Syn aggregation and sustained inflammation are mutually causal.

Under normal circumstances, apoptosis is crucial for normal nervous system development [11]. However, under pathological conditions, upregulation of proinflammatory cytokines, such as TNFα and IL6, can sensitize cells in the central nervous system to necroptosis [11]. Our study demonstrated that the inhibition or silencing of MLKL in mouse primary neuronal cells and human induced pluripotent stem cell (iPSC)-derived midbrain organoids (hMOs) protected them from α-Syn PFFs-triggered cell death. Furthermore, IL6 is reported to be elevated in serum samples from patients with PD in an age-dependent manner [39]. Here, we found that IL6 and MCP1 levels were reduced in cells lacking p-MLKL expression, as well as in Tg-*Mlkl*^*−/−*^ mice (Figs. 1 and 4). In addition, necroptosis can be induced in microglia and astrocytes under neurodegenerative conditions, and oxidative stress may trigger necroptosis and contribute to the pathogenesis of PD [45–47]. Although iNOS is not typically or is minimally expressed in the brain, its expression increases in the glia under pathological conditions [48]. Consistently, the glia was suppressed in the Tg*-Mlkl*^*−/−*^ mice. Also, knockout of MLKL or inhibiting its activity reduced the expression level of iNOS in PD mice’s brains or 6-OHDA plus TNFα-treated cells (Figs. 1 and 3). Overall, we propose that MLKL KO reduced the glia activation-triggered oxidative stress productions of iNOS and multiple proinflammatory cytokines.

Furthermore, we analyzed 12,029 single-cell transcriptomes across the Tg-*Mlkl*^*+/+*^ and Tg-*Mlkl*^*−/−*^ mice to gain a global insight into the biological function of MLKL in PD progression. The single-cell data confirmed a neuroprotective role for MLKL deficiency in the PD mouse model, as well as a dampening of the inflammatory response (Figs. 6, 7 and 8). The Tg-*Mlkl*^*−/−*^ microglia displayed lower mRNA. We identified 80 common mRNAs that exhibited differential expression between Tg-*Mlkl*^*+/+*^ and Tg-*Mlkl*^*−/−*^ mice in both neuronal, microglial, and astrocyte clusters (Fig. 6). Notably, the mRNA and protein levels of PTGDS were significantly decreased in the Tg-*Mlkl*^*−/−*^ group, which were confirmed through qPCR and IHC assays (Fig. 7). PTGDS is an enzyme that plays a role in the production of prostaglandin D2, a lipid mediator that regulates sleep [49]. Sleep disturbances are common in PD and can significantly impact patients’ quality of life [50]. Furthermore, PTGDS-immunoreactive isoforms have been observed in many neurodegenerative disorders, including AD and PD [51]. Based on these findings, we suggest that changes in PTGDS expression may be important in the pathogenesis of PD and that its function may contribute to the impact of MLKL deficiency on the progression of Parkinsonian traits. In addition, we identified upregulated common genes that may be involved in MLKL function in PD, such as *Xist*. The long non-coding RNA XIST (X-inactive specific transcripts) is derived from the XIST gene and is a key regulator of cell proliferation and differentiation [52]. A recent study demonstrated that lncRNA XIST sponges miR-199a-3P, thereby enhancing the expression of Sp1 and LRRK2, which accelerates PD progression [53]. Further research is necessary to elucidate the action mechanism of these genes in PD.

In summary, our findings suggest that MLKL KO reduces dopaminergic neuron loss and alleviates motor dysfunction in the A53T transgenic mouse model. Furthermore, MLKL plays a crucial role in the pathogenesis of PD by regulating inflammatory responses and oxidative stress in glial cells. Thus, our study provides new insights into the biological function of MLKL in PD progression and highlights the potential of MLKL as a therapeutic target for treating PD.

## Materials and methods

### Mice

The C57BL/6 mice with an *Mlkl*^*−/−*^ genotype were generated by Dr. Haibing Zhang at SIBS (Shanghai, China) using TALEN technology as described previously. Tail clips were extracted from offspring, and the primers used for MLKL genotyping were forward: 5′-CAGCACAAATCCCATCCACTC-3′ and reverse: 5′-TAAACCTGAAGCAGCAGCAAC-3′. The human *SNCA* A53T transgenic (Tg) mice were generated at the Central South University (Changsha, China). The primers used for Tg genotyping were forward: 5′-GGCAGAAGCAGCAGGAAAGAC-3′ and reverse: 5′-GGGCTCCTTCTTCATTCTTGC-3′. All animal experiments were performed in accordance with the NIH Guide for the Care and Use of Laboratory Animals, with the approval of the Scientific Investigation Board of the School of Life Sciences, Fudan University (2019-JS-011). C57BL/6 mice were obtained from the SLAC Laboratory Animal Co. (Shanghai, China). Mutant alleles were backcrossed with C57BL/6 mice for more than six generations. Mice used in the experiments were homozygotes at 11 to 12 months of age and 28 to 32 g of weight.

### Cell culture and treatment

Primary MEF and SH-SY5Y cells were cultured in the high-glucose DMEM (Hyclone) supplemented with 10% (v/v) fetal bovine serum (Gibco). All cells were grown at 37 °C in a 5% CO_2_ incubator (Thermo Fisher, USA). PolyJet (Signage, USA) was used for the transfection of corresponding plasmids into cells. For the 6-OHDA-induced cell death experiment, cells were plated into a 96-well plate before treatment with 50 µM of 6-OHDA, 2.5 ng/ml of TNF-α, and 5 µM necrosulfonamide (NSA). Cell viability was analyzed using an Enhanced Cell Counting Kit-8 (CCK8) Assay Kit (Beyotime, China) according to the manufacturer’s instructions. Briefly, 5 × 10^3^ cells were seeded in 96-well plates. After 12 h, the cells were treated with corresponding reagents for 24 h. The medium was then changed to a medium containing 5% CCK8-reagent (Beyotime, China) after 24 h. The incubation was continued for one hour in the dark. Then, the absorbance was measured at a wavelength of 450 nm using a Spectra-Max M5 plate reader (Molecular Devices).

### α-Syn purification and α-Syn PFFs preparation

Full-length human α-synuclein cDNA was cloned into the *Sca1* and *Xho1* restriction sites of the pSMT3 bacterial expression vector and expressed in BL21-CodonPlus (DE3)-RIL cells. Post-cultivation, bacterial cells were collected by centrifugation at 6000 rpm for 15 min at 4 °C and then resuspended in a high-salt buffer (comprising Tris-HCl pH 8.0, 100 mM NaCl, 10 mM imidazole pH 8.0, 5% glycerol, 4 mM MgCl_2_, and 2 mM β-mercaptoethanol). Cell disruption was achieved using a high-pressure homogenizer (JNBIO, China). The resulting supernatants were further processed through a Superdex200 16/600 column (GE Healthcare) using a gel-filtration buffer (20 mM Tris-HCl pH 7.4, 100 mM NaCl, 2 mM DTT). For α-Syn PFFs preparation, recombinant α-Syn was diluted to 5 mg/ml in PBS and incubated at 37 °C (1000 rpm) for 7 days. The successful fibrillization of α-Syn was confirmed by negative-staining electron microscopy and a thioflavin T-binding assay. The assembled α-Syn PFFs were aliquoted, sonicated (60 pulses at 10% power, 0.5 s per pulse) using a QSonica Microson XL-2000, and stored at -80 °C.

### Primary neuron culture and α-Syn PFFs treatment

Midbrain neuron cultures were derived from embryos of pregnant C57BL/6 mice (E16-E18). The isolation and culturing of primary neurons were carried out following a previously published protocol (https://www.nature.com/articles/nprot.2014.143#Sec12). Dissociated midbrain neurons were seeded onto poly-d-lysine-coated plates at a density of 100,000 cells/well in a 24-well plate using neurobasal media supplemented with B-27, N-2, 0.5 mM L-glutamine, penicillin, and streptomycin (Thermo Fisher, USA). At 7 days in vitro (DIV), neurons were treated with PBS, sonicated α-Syn PFFs (5 µg/ml), or NSA (5 µM). The culture medium was replaced every 3–4 days, with no additional α-Syn PFFs added post-treatment. After 14 days, the treated cells were harvested for subsequent experiments.

### Human midbrain organoids (hMOs) culture and cell treatment

To model Parkinson’s disease in vitro, we utilized human induced pluripotent stem cell (iPSC)-derived midbrain organoids (hMOs). These organoids were generated following a previously established protocol, which guides the differentiation of iPSCs into midbrain-specific neuronal cells [54]. The iPSCs were cultured and maintained in Essential 8 Medium on Matrigel-coated plates. For differentiation into midbrain organoids, the iPSC colonies were detached and reaggregated in low-attachment plates in a differentiation medium containing dual SMAD inhibitors. The culture medium was supplemented with midbrain-specific growth factors, including FGF8 and SHH, to promote midbrain patterning. The organoids were cultured for 8 weeks, allowing for the development of complex, midbrain-like structures.

The matured hMOs were subjected to three different treatment conditions. The first group was treated with α-synuclein preformed fibrils (PFFs) to induce Parkinson’s disease-like pathology. The second group received a combination treatment of PFFs and adeno-associated virus serotype 9 (AAV9) engineered shRNA to knock out MLKL gene expression (AAV9-shMLKL, shMLKL: GATCCTTGTTGAATACTTTGATGGTATCAAGAGTACCATCAAAGTATTCAACAATTTTTA). The control group was maintained without any treatment. For the PFFs treatment, the organoids were incubated with a PFF solution at a concentration of 5 µg/mL. The AAV9-mediated knock-out of MLKL was performed by infecting the organoids with AAV9 vectors at an MOI of 1000, 24 h prior to PFF treatment. Post-treatment, the organoids were maintained under standard culture conditions and monitored for an additional 14 days to assess the development of PD-related phenotypes and the impact of MLKL knock-out on these phenotypes.

### Reagents and antibodies

6-OHDA (HY-B1081), TNF-α (HY-P7090), and NSA (HY-100,573) were purchased from MCE. The antibodies used for western blot were anti-Alpha-synuclein (1:10000, ab138501, Abcam), anti-Alpha-synuclein (phospho S129) (1:5000, ab51253, Abcam), anti-β-actin (1:5000, ab8226, Abcam), anti-TH (1:1000, ab75875, Abcam), anti-MLKL (1:1000, AP14272B, abcepta), anti-MLKL (phospho S345) (1:1000, ab196436, Abcam), and anti-iNOS (1:1000, ab283655, Abcam). The primary antibodies for immunostaining were listed as follows: rabbit anti-Alpha-synuclein (phospho S129) (1:1000, ab51253, Abcam), rabbit anti-TH (1:400, ab75875, Abcam), mouse anti-GFAP (1:2000, ab4648, Abcam), rabbit anti-Iba1 (1:2000, ab178846, Abcam), rabbit anti-CD11b (1:5000, ab133357, Abcam), and rabbit anti-PTGDS (1:500, 10754-2-AP, Proteintech). Fluorescent secondary antibodies were purchased from Invitrogen (USA) and were used in a 1:2000 dilution. Immunohistochemical secondary antibodies were purchased from BOSTER (SA2002) and were used in a 1:200 dilution.

### Protein preparation and immunoblotting analysis

Mouse brain tissue, including corpus striatum, substantia nigra, and cortex areas, were homogenized and prepared in a lysis buffer (50 mM Tris (pH 7.4), 150 mM NaCl, 1% Triton X-100, 1% sodium deoxycholate, 0.1% SDS, sodium orthovanadate, sodium fluoride, EDTA, leupeptin, phosphatase inhibitor mixture (100X, Beyotime), and the complete protease inhibitor mixture (100X, Beyotime)) using a bead mill homogenizer (70 Hz, 3 min). After homogenization, samples were rotated at 4 °C for 1 h for complete lysis. The homogenate was then centrifuged at 15,000 x g for 1 h, and the supernatants were used for further analysis. Samples were separated using SDS-polyacrylamide gels and transferred onto nitrocellulose membranes. The membranes were blocked with 5% non-fat milk in TBS-T (Tris-buffered saline with 0.1% Tween-20) for 1 h, probed using corresponding primary antibodies, and incubated with appropriate HRP-conjugated secondary antibodies. The bands were visualized by the ECL substrate.

### Immunofluorescence staining, immunohistochemistry staining, and confocal analysis

For immunofluorescence analysis, the 40 μm free-floating serial coronal frozen sections of mouse tissues were incubated for 30 min at 37 °C before being blocked with a 5% goat serum (Gibco) for 30 min at 37 °C. Next, sections were incubated overnight at 4 °C with primary antibodies. Finally, after rinsing three times with PBS, sections were incubated with species-specific secondary antibodies and were visualized under the fluorescence microscopy (Lecia TCS SP8, Germany).

Sections were deparaffinized in xylene for immunohistochemistry analysis and rehydrated through a decreasing ethanol gradient. Endogenous peroxidase activity was blocked by 3% H_2_O_2_ for 10 min. Heat-mediated antigen retrieval was performed by placing slides in a sodium citrate buffer (pH 6.0) and was heated twice in a microwave. Next, the sections were blocked in a 5% goat serum (Boster, China) at 37°C for 45 min. Slices were incubated with primary antibodies at 4°C overnight, washed three times in PBS, and incubated with corresponding biotin-labeled secondary antibodies for 40 minutes, then washed in PBS and incubated with a SABC (streptavidin-biotinylated complex) for 25 min. Immunostaining was achieved using 3’,3’-diaminobenzidine tetrahydrochloride (DAB/H_2_O_2_). All tissue sections were counterstained with hematoxylin.

### ELISA

The proinflammatory response was determined in mice serum and cell culture supernatant using the Cytokine & Chemokine 36-Plex Mouse ProcartaPlex™ Panel 1 A for 24 cytokines (IL-1β, IL-4, IL-6, IL-10, IL-15, IL-17 A, IL-27, IL-28, IL-22, IL-23, IL-31, MIP-1β, MIP-2, TNF-α, IFNγ, IP-10, MCP-1, CCL3, CCL5, CXCL1, CXCL2, CXCL5, GM-CSF, and G-CSF) following the protocol provided by the supplier (ThermoFisher, USA). Briefly, 50 µl of the diluted sample, calibrator, or control was added per well. The plate was sealed with an adhesive plate seal and incubated at room temperature with shaking for 2 h. Then, the plate was washed three times and the detection antibody was added with shaking for 2 h. Finally, the plate was washed and the read buffer was added. Signals were measured on a SECTOR Imager 2400 reader (MSD, USA).

### Behavioral tests in mice

The behavioral experiments were performed during the light cycle (10:00 am to 17:00 pm) on male and female mice of both genotypes. On the test day, mice were transferred to the testing room and allowed a resting time of 60 min before testing.

Rotarod test. Mice were placed into a rotating rod (Model LE8500, Panlab SL). All mice were trained for three consecutive days before testing on the fourth day. On day 1, mice were trained with a fixed speed of 4 rpm for 300 s on the rotarod apparatus four times. On days 2, 3, and 4, mice were trained and tested with an increasing speed from 4 to 40 rpm four times. After every trial, the rods were cleaned with 70% ethanol to avoid potential odor cues. The time a mouse spent on the rod before falling off was recorded as latency. The mean value of the three longest latencies was used for analysis.

Pole test. The pole test was implemented to test the motor coordination of mice. Mice were placed facing upward at the top of a vertical pole (60 cm high, 0.6 cm in diameter, wrapped with gauze to prevent slipping) and given 180 s to change orientation and descend the pole. The base of the pole was covered with bedding as protection for mice from injury. Failure to descend or fall from the pole was assigned 180 s. All mice were trained for two consecutive days before testing on the third day. Three trial times were performed for each mouse, and the average latency to descend was determined.

Tail suspension test. The tail suspension test was used to evaluate the depressive-like symptoms of mice. During the test, each mouse was suspended by taping its tail to a horizontal bar with an upside-down position. The mouse head was about 15 cm away from the bottom of the tail suspension box. The trial was conducted for 6 min under video recording, and the amount of immobility time was scored. It was repeated two times to exclude for further analysis when tail crawling was observed (> 10% of the total time).

Open-field test. The mice’s general locomotor activity and anxiety-like behaviors were measured in an open field box (40 cm × 40 cm × 40 cm). Each mouse was placed in the central area and the behaviors, including total traveled distances, number of entering the center area, and time spent in the center area were recorded and evaluated for 10 min with a video-imaging system (EthoVisionXT; Noldus Information Technology, The Netherlands).

Elevated plus-maze test (EPMT). Anxiety-like behavior was measured using the elevated plus-maze. The elevated plus-maze consisted of two open and two closed arms (30 cm in length, 5 cm in width). The maze was made of non-reflecting light grey PVC. Each mouse was placed in the central area of the maze facing one of the open arms. The duration of the mice in each arm, the number of the mice entering open arms, and the total traveled distance were continuously assessed for 5 min using EthoVisionXT.

### Nuclei isolation from frozen mouse brain tissue

The protocol for isolating nuclei from frozen brain tissue was described before [55]. All procedures were carried out on the ice or at 4 °C. Briefly, the frozen substantia nigra brain tissues were homogenized in 2 ml homogenization buffer (10 mM Tris-HCl, pH 8.0, 25 mM KCl, 5 mM MgCl_2_, 0.1% Triton X-100, 250 mM sucrose, 0.5 U/µl SuperRNase inhibitor, and 0.5 U/µl RNAse inhibitor), using a Wheaton Dounce Tissue Grinder (10 strokes with the loose pestle and 15 strokes with the tight pestle). Then, the tissue homogenate was overlaid onto 10% iodixanol and centrifuged with an SW32 rotor at 10,000 g for 20 min at 4 °C. After ultracentrifugation, the supernatant was aspirated and discarded, and the remaining pellet was resuspended in 0.5% BSA plus 50 ng/ul DAPI for nuclei isolation using a BD AriaFusion cell sorter (BD Biosciences).

### Single-cell RNA sequencing and data analysis

Nuclei were sequenced using droplet-based single-nucleus RNA sequencing (snRNA-seq) using the Chromium system (10x Genomics) to generate libraries for sequencing on the Illumina NovaSeq platform with manufacturer’s instructions.

Data analysis was performed in R using the Seurat version 4.0.2, following standard recommendations. Specifically, data were first filtered for quality, so only genes expressed in more than five cells and cells with more than 200 genes and a mitochondrial transcript ratio of less than 20% were kept. Then normalization and scaling were performed with default parameters. Finally, dimension reduction was carried out by first identifying the so-called highly variable genes using default parameters. Then the Principle Component (PC) Analysis was applied to those genes to obtain the top 20 most significant PCs. These PCs were then used for cell visualization with UMAP embedding. They were further used to cluster the cells with a resolution of 0.8. The resulting clusters were annotated using known marker genes for 7 cell types, including oligodendrocyte, astrocyte, neuron, microglia, oligodendrocyte precursor, type II spiral ganglion neuron, and Bergman glial cell (Fig. 5). To identify neuron, microglia, and astrocyte subpopulations, we re-clustered each primary cluster with a resolution of 0.6.

Differential expression (DE) analysis for the indicated clusters (Figs. 5 and 6) was performed using the FindMarkers function within Seurat. Significant DE genes were identified based on the Wilcoxon rank-sum test with a Benjamini-Hochberg corrected *P*-value < 0.05 and the log2 fold change greater than 0.01.

### RNA isolation and expression analysis of individual candidate genes

Total RNA isolation from brain tissue was performed using TRIzol reagent (Invitrogen) as per the manufacturer’s instructions. For qRT-PCR assay, cell RNAs were transcribed into cDNAs using the PrimeScript™ 1st Strand cDNA Synthesis Kit (Takara). Reactions were done in duplicate using SYBR Select Master Mix (Life Technologies). The prime sequences are as follows: Ptgds (Forward primer sequence: 5’-TCGCCTCCAACTCAAGCTGGTT-3’, Reverse primer sequence: 5’-CCATGATCTTGGTCTCACACTGG-3’). Quantitative reverse transcriptase PCR was performed by the Life Q7 Real-Time PCR system (J1502336). Expression levels were normalized to GAPDH.

### GO enrichment analysis

We used the R package cluster Profiler to perform statistical overrepresentation analysis for the DE genes to identify enriched gene ontology (GO) categories. The hypergeometric test with FDR correction was applied to the Biological Processes ontology. A GO term was considered significantly enriched if the FDR-adjusted *P* value was < 0.05. The GO enrichment analysis was performed on the same set of DE genes, ‘Tg-*Mlkl*^*−/−*^ vs. Tg-*Mlkl*^*+/+*^’.

### Statistical analysis

Each experiment was performed at least three times. All experiment data were analyzed using GraphPad Prism 8.0 (GraphPad software Inc. USA) and were presented as the mean ± SD. Statistical analysis was performed using one-way ANOVA or two-way ANOVA. A value of *P < 0.05* was considered statistically significant.

### Electronic supplementary material

Below is the link to the electronic supplementary material.

Supplementary Material 1

## Data Availability

All single-cell RNA sequencing data are available from the Gene Expression Omnibus (GEO) under the accession number GSE197679. All data needed to evaluate the conclusions in the paper are present in the paper and/or the Supplementary Materials. Additional data related to this paper may be requested from the corresponding author.

## Abbreviations

6-OHDA: 6-Hydroxydopamine Hydrobromide
α-Syn: α-synuclein
AD: Alzheimer’s Disease
ALS: Amyotrophic Lateral Sclerosis
CNS: Central Nervous System
DA: Dopaminergic
DEGs: Differential expression of genes
DLB: Dementia with Lewy Bodies
ELISA: Enzyme-linked Immunosorbent Assay
EPMT: Elevated Plus-Maze Test
GFAP: Glial Fibrillary Acidic Protein
GO: Gene Ontology
ILBD: Incidental Lewy Bodies Disease
iNOS: Inducible Nitric Oxide Synthase
KO: Knockout
MEFs: Mouse Embryonic Fibroblasts
MLKL: Mixed-lineage Kinase Domain-like
MS: Multiple Sclerosis
MSA: Multiple System Atrophy
Nec-1: Necrostatin-1
NSA: Necrosulfonamide
PD: Parkinson’s Disease
PDD: Parkinson Disease Dementia
PFFs: Preformed Fibrils
PTGDS: Prostaglandin D synthase
RIPK1: Receptor-Interacting Protein Kinase 1
RIPK3: Receptor-Interacting Protein Kinase 3
scRNA-seq: Single-cell RNA-seq
SN: Substantia Nigra
SNpc: Substantia Nigra Pars Compacta
TH: Tyrosine Hydroxylase
TNF-α: Tumor Necrosis Factor-α
UMAP: Uniform Manifold Approximation and Projection
WT: Wild-Type
