# Supplementary material for: MLKL deficiency alleviates neuroinflammation and motor deficits in the α-synuclein transgenic mouse model of Parkinson’s disease
Source: Mol Neurodegener. 2023 Dec 1;18:94. doi: 10.1186/s13024-023-00686-5 (PMC10693130; doi:10.1186/s13024-023-00686-5)
Supplement: Supplementary file 1 — Supplementary Material 1 [file 13024_2023_686_MOESM1_ESM.docx]

**Supplementary Figures**

**
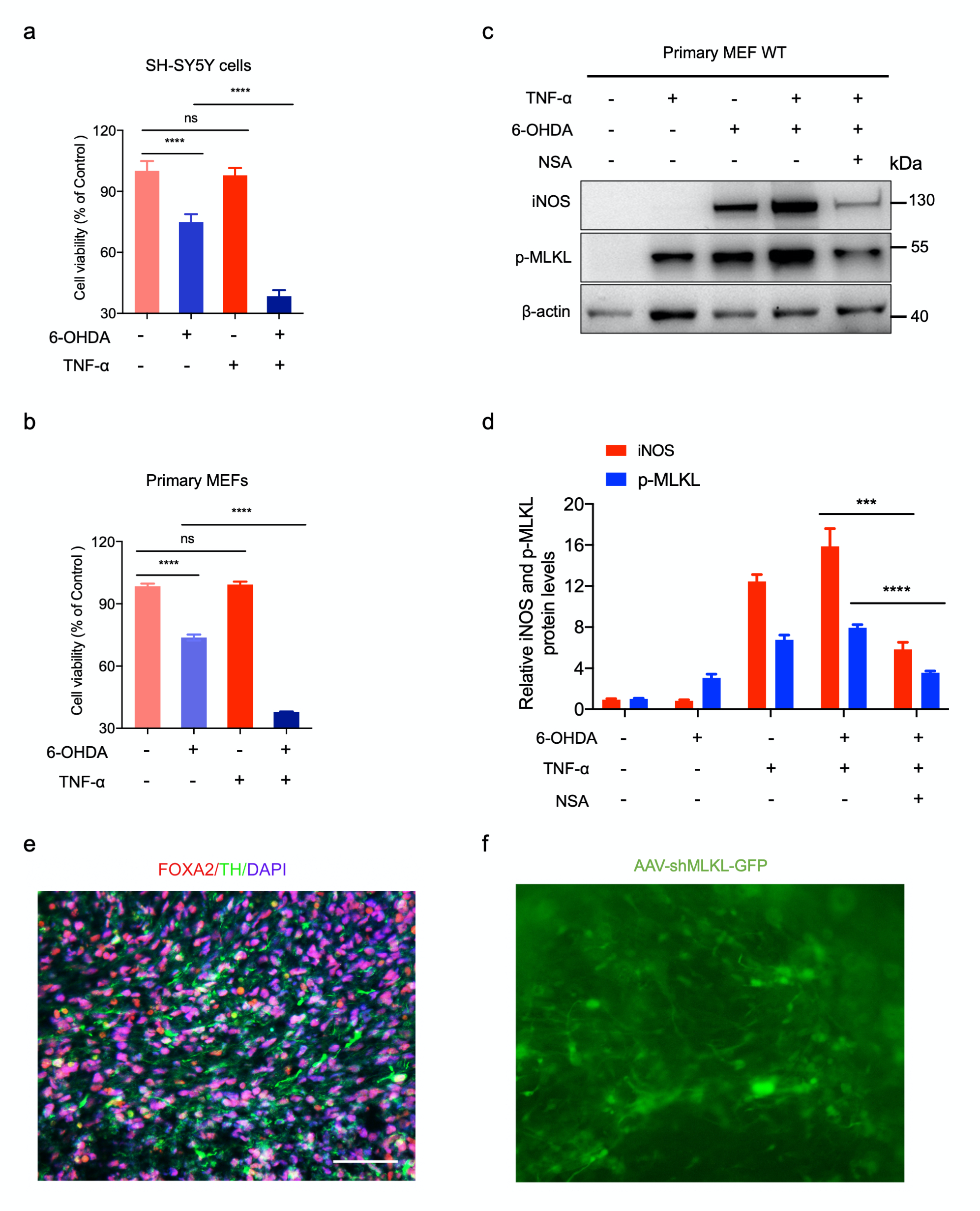
Figure S1**. TNF-α sensitizes different cells to 6-OHDA or toxic α-Syn PFFs induced cell death in PD stress conditions, related to **Figure 1**. **a.** SH-SY5Y cells were co-treated with or without 6-OHDA or/and TNF-α for 24 h. Cell viability was analyzed by the CCK8 assay. **b**. Primary MEF cells were co-treated with or without 6-OHDA or/and TNF-α for 24 h. Cell viability was analyzed by the CCK8 assay. **c-d**. Primary MEF cells were treated with or without 6-OHDA or/and TNF-α for 24 h. Then western blot analysis was performed to measure iNOS, p-MLKL, and β-actin levels (**c**). The corresponding quantification results were shown in **d**. **e**. Human iPSC-derived midbrain organoids (hMOs) were stained with FOXA2, TH, and DAPI. Scale bars, 100 μm. **f**. iPSC cells were transfected by aav-shMLKL-GFP. All data are representative of three independent experiments. The error bars represented the standard deviations (SD). *** *p* < 0.001, **** *p* < 0.0001, ns, no significance.

**
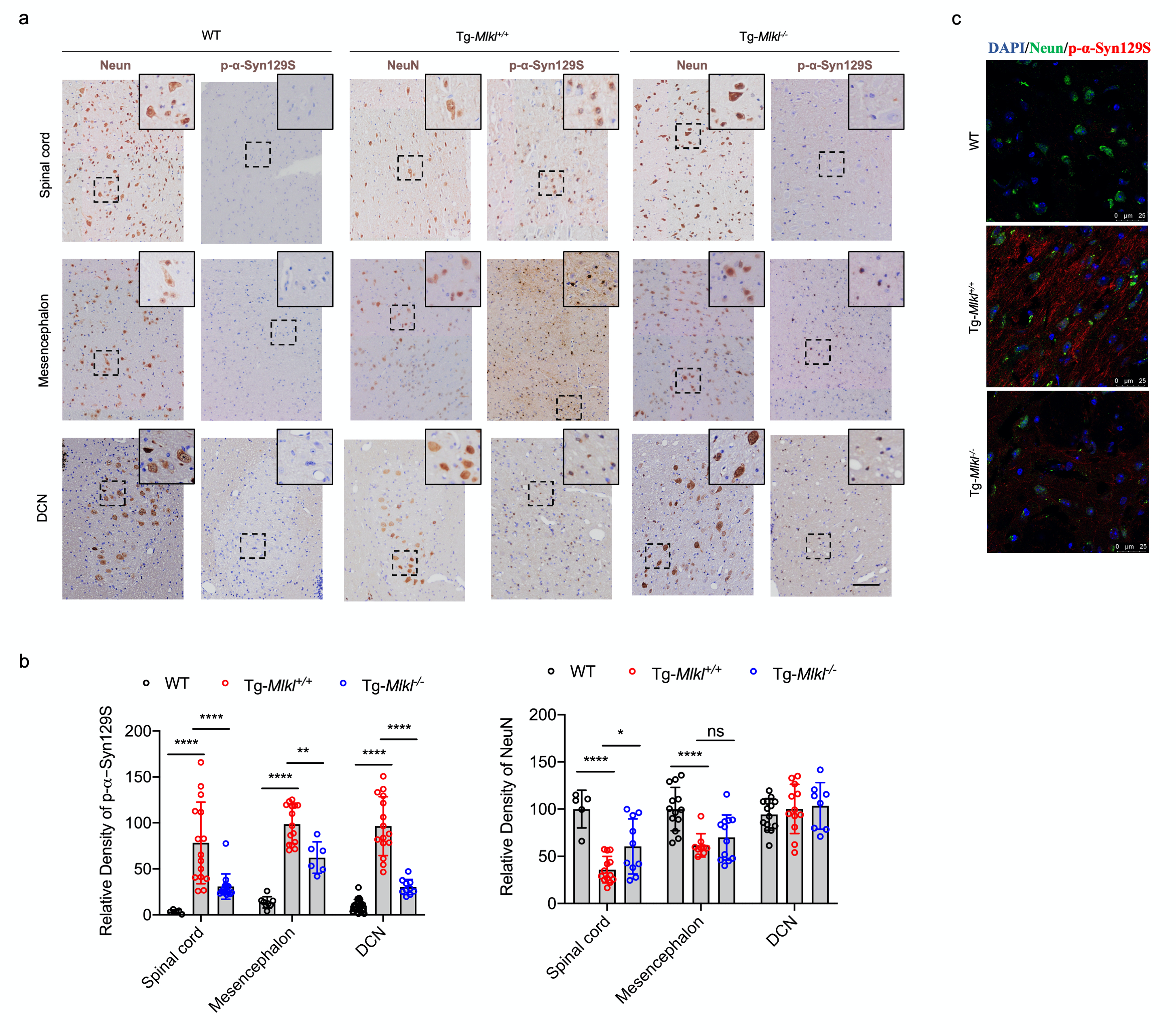
Figure S2.** MLKL deficiency mitigates dopaminergic neuron loss and reduces p-α-synuclein in A53T transgenic mice, related to **Figure 3**. **a-b**. Representative immunostaining images for phosphorylated α-Synuclein (p-α-Syn129S) in the spinal cord, mesencephalon (surrounding the substantia nigra) and dorsal cochlear nucleus (DCN) regions of WT, Tg-*Mlkl^+/+^*, and Tg-*Mlkl^-/-^* mice (**a**). Scale bars are set at 100 μm. Detailed views (solid rectangles) are extracted from the indicated areas (dashed rectangles) in the spinal and DCN regions. The corresponding quantification results of NeuN and p-α-Syn129S are presented in **b**. **c**. Representative immunofluorescence images from the cortex region of WT, Tg-*Mlkl^+/+^*, and Tg-*Mlkl^-/-^* mice's frozen brain sections. Staining includes DAPI (blue) for nuclear signal identification, anti-NeuN (green), and anti-p-α-Syn antibody (red). Scale bars measure 25 μm. All data are representative of three independent experiments. The error bars represented the standard deviations (SD). * *p* < 0.05, ** *p* < 0.01, **** *p* < 0.0001, ns, no significance.


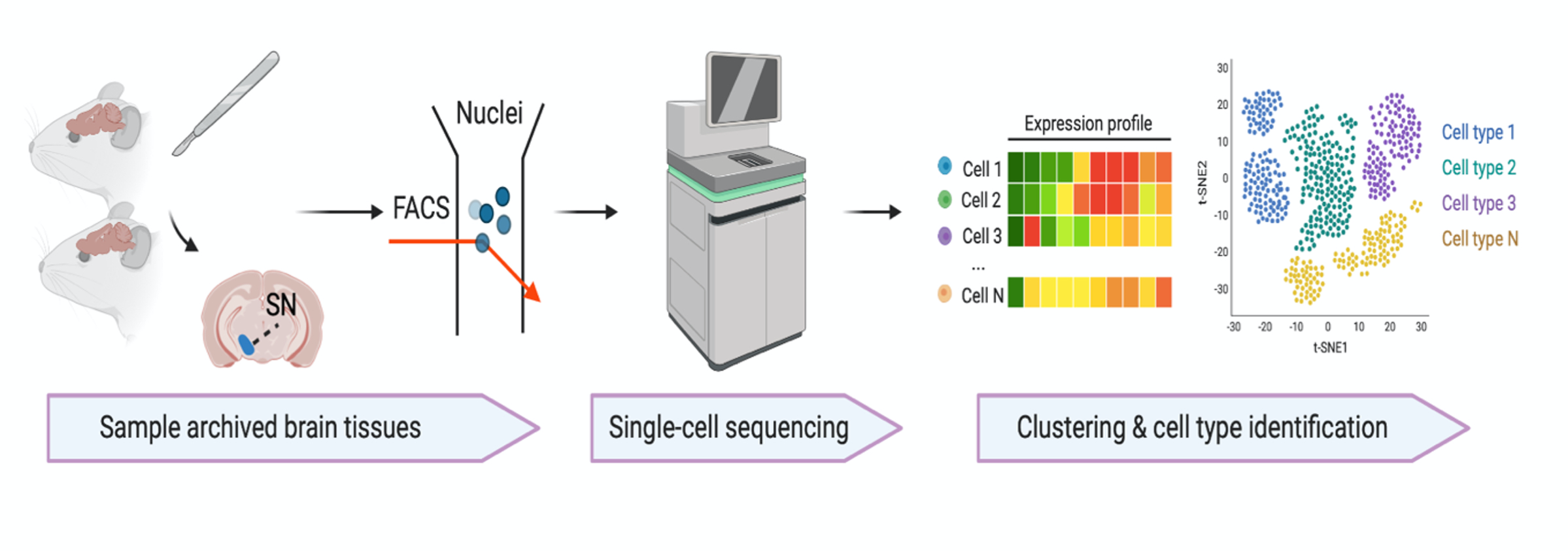


**Figure S3.** Schematic graph of the nuclei isolation and single-cell RNA-seq analysis workflow.


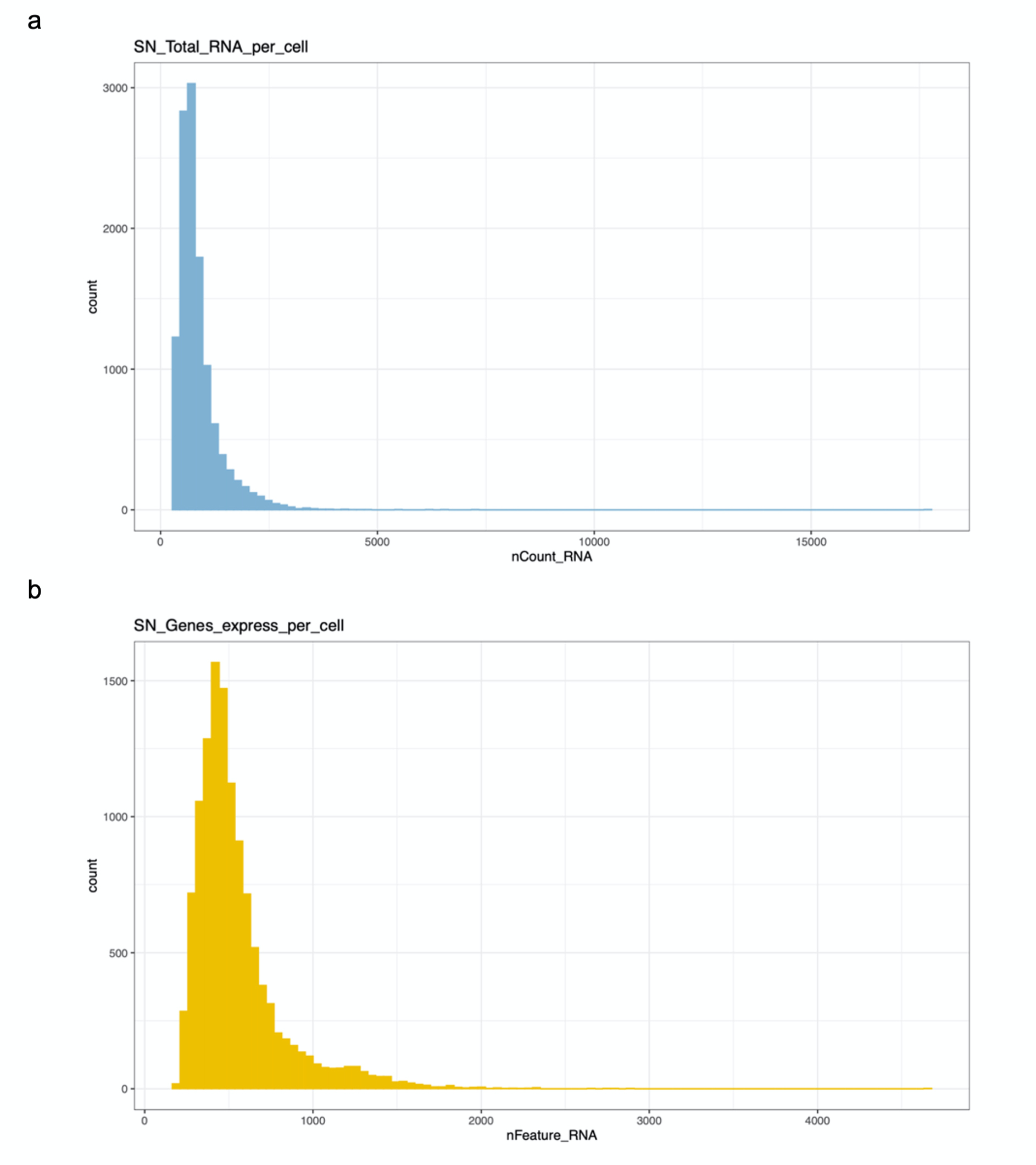


**Figure S4**. Quality control used for filtering single-cell RNA-seq data, related to **Figure** **6-8**. **a.** Histogram showing the final distribution of total RNA per cell. **b.** Histogram showing the final distribution of the number of genes expressed per cell.

**
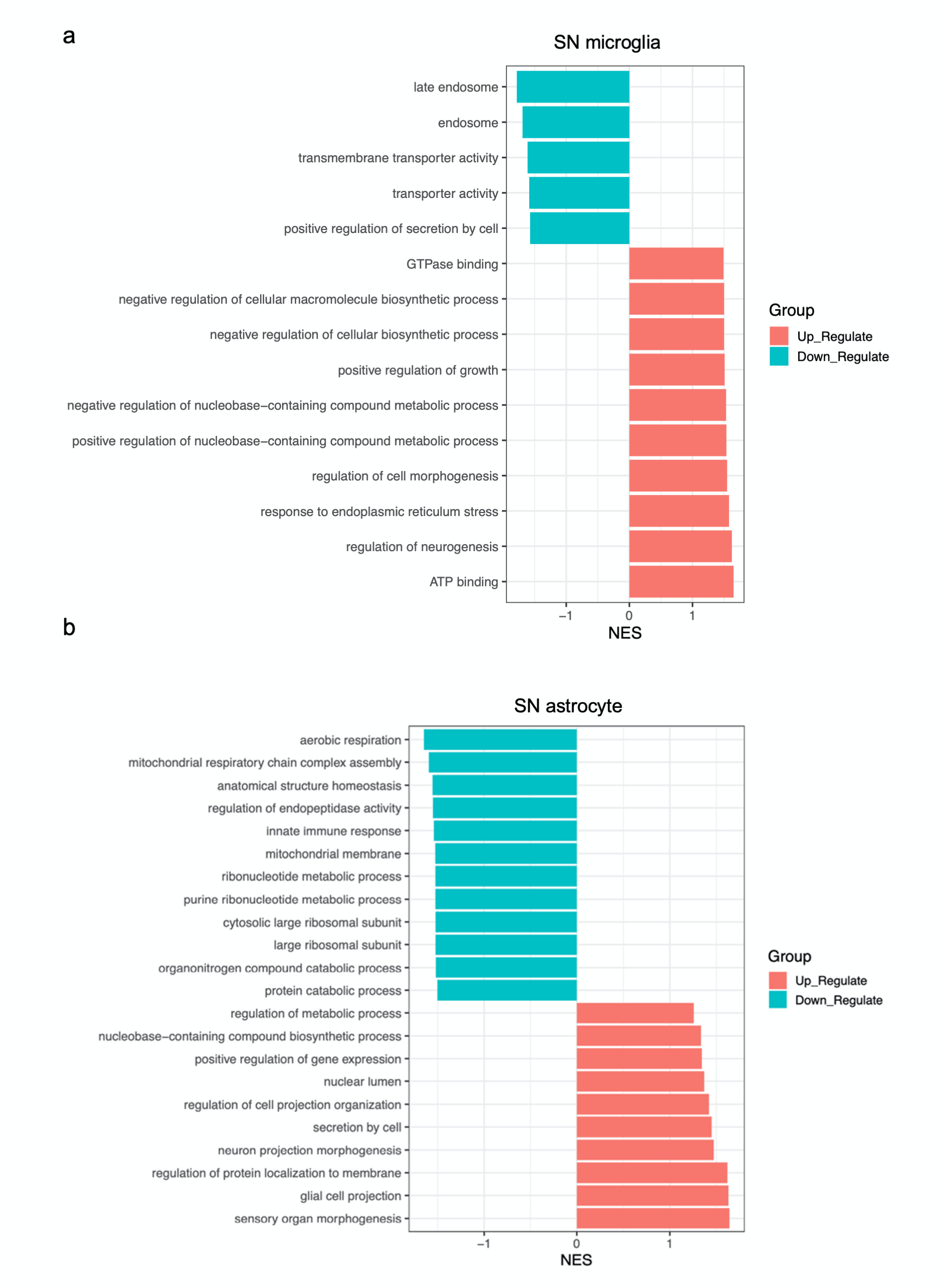
**

**Figure S5**. Top biological pathways enriched for DEGs identified across microglia (**a**) and astrocyte clusters (**b**), related to **Figure 6**.


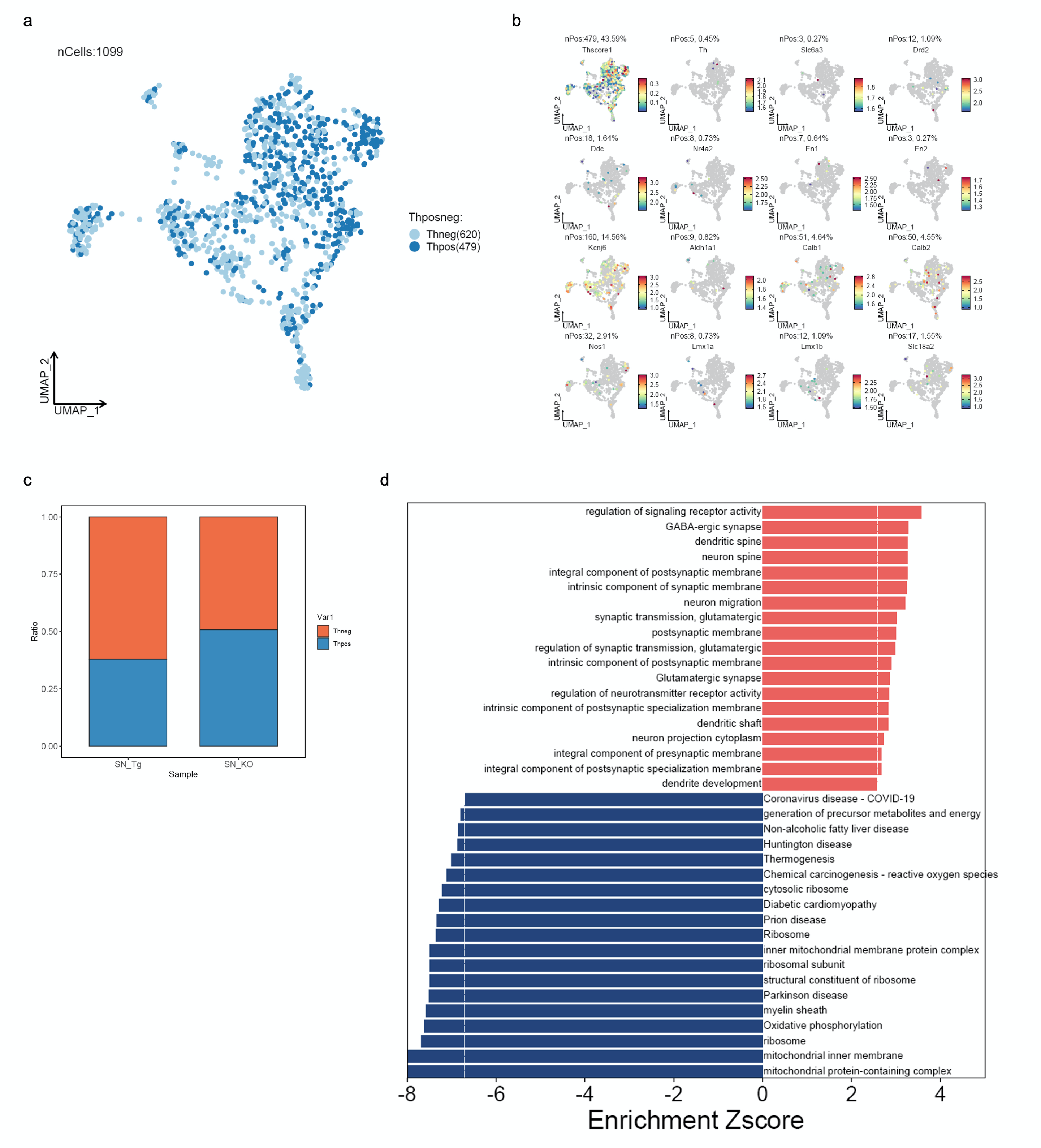


**Figure S6**. Subcluster-specific analysis revealing dopaminergic neuron subclusters within the neuronal cluster, related to **Figure 6**. **a**. UMAP visualization of subclusters of Thpos and Thneg cells. **b**. A feature plot is provided with highlighting 15 markers, including *Th*, *Slc6a3*, *Drd2*, *Ddc*, *Nr4a2*, *En1*, *En2*, *Kcnj6*, *Aldh1a1*, *Calb1*, *Calb2*, *Nos1*, *Lmx1a*, *Lmx1b,* and *Slc18a2* utilized for identifying the Thpos (positive) and Thneg (negative) clusters. **c**. The frequency distribution of each subcluster between Tg-*Mlkl^+/+^* and Tg-*Mlkl^-/-^* mice is depicted, offering insights into the distribution and differences in these subclusters across the two genotypes. **d**, Top biological pathways enriched for DEGs were identified across Thpos cells in the SN region.

**
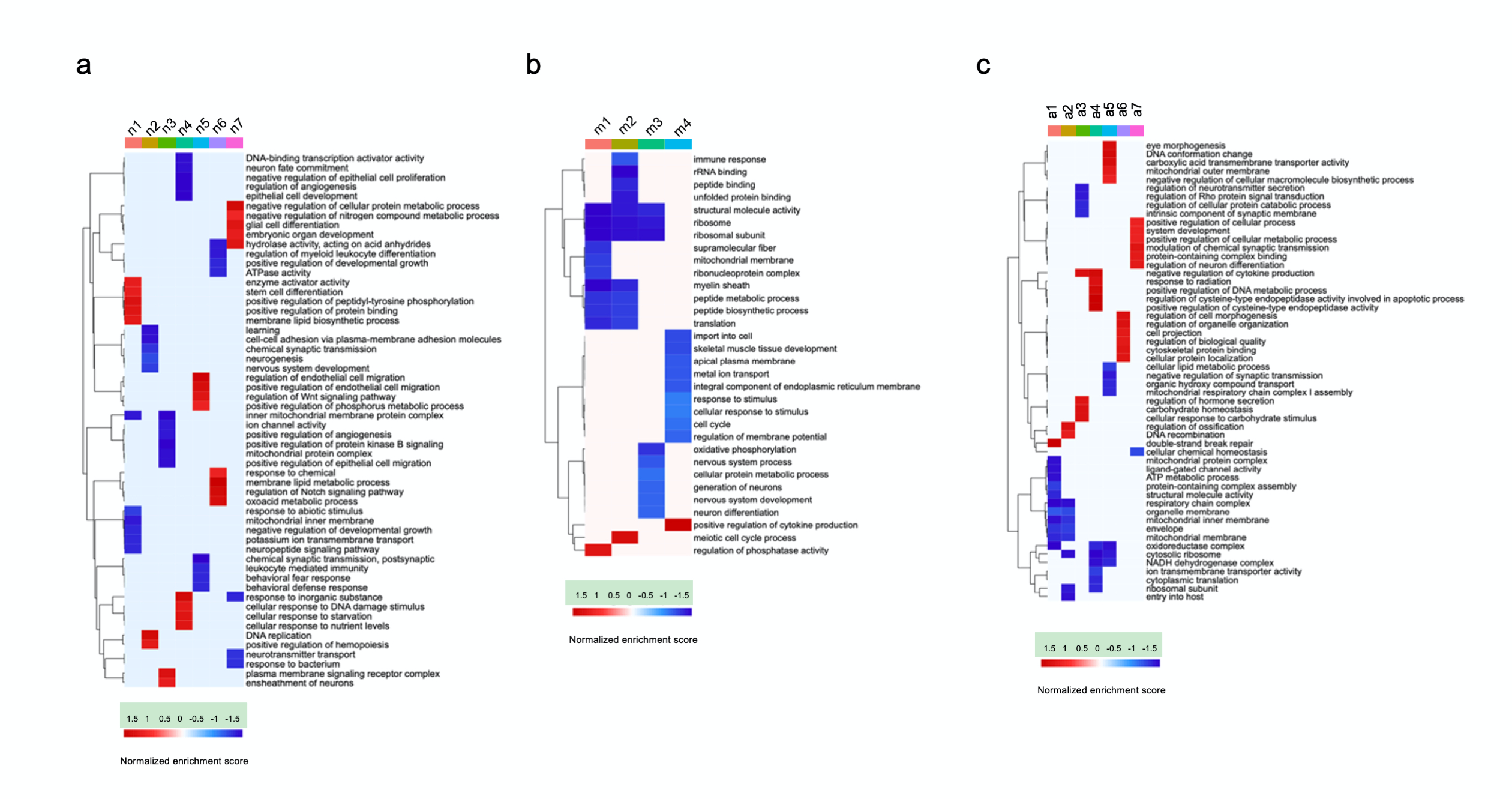
Figure S7.** The gene set enrichment analyses (GSEA) of subcluster-specific differential expressions in neurons, microglia, and astrocytes were colored by statistical significance. The normalized enrichment scores for selected gene ontologies were shown in each cell subcluster, related to **Figure 8**.
